# EUP: Enhanced cross-species prediction of ubiquitination sites via a conditional variational autoencoder network based on ESM2

**DOI:** 10.1371/journal.pcbi.1013268

**Published:** 2025-07-16

**Authors:** Junhao Liu, Zeyu Luo, Rui Wang, Xin Li, Yawen Sun, Zongqing Chen, Yu-Juan Zhang

**Affiliations:** 1 College of Life Science, Chongqing Normal University, Chongqing, P.R. China; 2 School of Mathematical Sciences; Chongqing Key Lab of Cognitive Intelligence and Intelligent Finance, Chongqing Normal University, Chongqing, P.R. China; Huazhong University of Science and Technology, CHINA

## Abstract

Ubiquitination is critical in biomedical research. Predicting ubiquitination sites based on deep learning model have advanced the study of ubiquitination. However, traditional supervised model limits in the scenarios where labels are scarcity across species. To address this issue, we introduce EUP, an online webserver for ubiquitination prediction and model interpretation for multi-species. EUP is constructed by extracting lysine site-dependent features from pretrained language model ESM2. Then, utilizing conditional variational inference to reduce the ESM2 features to a lower-dimensional latent representation. By constructing downstream models built on this latent feature representation, EUP exhibited superior performance in predicting ubiquitination sites across species, while maintaining low inference latency. Furthermore, key features for predicting ubiquitination sites were identified across animals, plants, and microbes. The identification of shared key features that capture evolutionarily conserved traits enhances the interpretability of the EUP model for ubiquitination prediction. EUP is free and available at (https://eup.aibtit.com/).

## Author summary

We present EUP, an AI-powered web-based tool designed to predict ubiquitination sites on protein sequences. Ubiquitination is a critical regulatory process in cells, where small ubiquitin molecules are attached to specific lysine residues to control protein stability and function. Identifying these modification sites is essential for understanding cellular mechanisms, but experimental detection is time-consuming and often unavailable for many non-model organisms. To address this challenge, EUP leverages a pretrained protein language model to extract informative features from sequences and applies variational inference to predict likely ubiquitination sites. The tool supports prediction across animals, plants, and microbes, and highlights both conserved and species-specific patterns. This provides users with interpretable insights into how ubiquitination may vary across evolution. EUP is freely available at (https://eup.aibtit.com) and offers a practical solution for researchers studying protein regulation across diverse biological systems.

## 1. Introduction

Ubiquitination is a crucial post-translational modification in biological organisms, involving the attachment of ubiquitin molecules to target proteins. This modification acts as a degradation signal and plays a vital role in regulating protein function, localization, and interactions [1,2]. The conjugation of ubiquitin to specific lysine site residues on the target protein determines the specificity of ubiquitin attachment, thereby influencing the protein’s fate, function, and interactions within the cell [3].

One question arises in highly accurate identifying the ubiquitination or non- ubiquitination across all lysine residues within amino acid sequence. Traditional experiment identifies protein ubiquitination employs two key methods: immunoprecipitation to detect ubiquitination and assays to measure E3 ligase activity [4,5]. Experiments provided a crucial pathway for recognizing ubiquitination site and understanding protein modifications, However, these methods have several limitations, including being time-consuming, resource-intensive, bothered with unstable experimental outcomes, and facing challenges with uncontrolled protein degradation [6,7]. In comparison, benefit to the emergence of numerous highly quality ubiquitination site datasets [8–10], machine-learning driven methods have been harnessed to address these challenges. By constructing models to predict binary classification of lysine residues, these methods achieve a relatively high accurate identification of ubiquitination site [8] in some cases.

For predicting protein ubiquitination sites, most models have been constructed using small-scale architectures and rely on feature extraction based on prior knowledge. For instance, one approach utilizes one-hot encoded amino acid features combined with a Convolutional Neural Network (CNN) to develop a deep learning-based model for ubiquitination site prediction [6]. Another method leverages manually engineered physicochemical properties of proteins and employs a Support Vector Machine (SVM) classifier to create a model specifically designed for ubiquitination site prediction in *Arabidopsis thaliana* [11].

However, the heavily rely on hand-crafted features and the limited number of trainable parameters in the models [6,11], may restrict their generalization performance. Specifically, both previous reports and our experiments have shown that these models exhibit limitations when evaluated on more diverse datasets, particularly those with species variations or noisier data [8,12]. A key challenge lies in the imbalance of data, where the number of non-ubiquitination sites far exceeds ubiquitination sites in amino acid sequences, making it difficult to train class-balanced prediction models. Even the most rigorously reviewed publications in this field [12] fail to adequately address the need for balanced data division or proper de-noising of non-ubiquitination data [13,14]. A more pressing concern is that almost all available methods or protocols are either not user-friendly as web servers or are currently inaccessible [12,14]. This raises a critical question: how many of the bioinformatics tools reported in the literature are genuinely useful and accessible for ubiquitination-related experiments and discoveries in the field.

To address the above question, we first deployed ESM2 (Evolutionary scale model), a large pretrained protein language models, to extract feature in each lysine sites. Compared to traditional window-driven methods [11,15], ESM2 captures more information related to potential biology structure, function, and even evolutionary information [16], leading to better feature representation. These lysine site feature extracted by ESM2, when combined with an attention mechanism, are naturally adept at compressing global information across the entire sequence while remaining local information [17,18].

Second, we explored several methods including de-homology, random under-sampling in most classes, combining cVAE (a variant mode of variational-autoencoder) and NCR (Neighbourhood Cleaning Rule) methods to perform data denoising and construct more balanced datasets. Finally, based on strict cleaned datasets and ESM2 lysine site feature, we developed a powerful in generation model namely EUP (ESM2 based ubiquitination sites prediction protocol) that performs well across species. Through rigorous experiments and evaluations on various processed datasets and different species, we demonstrated that EUP data cleaning protocol and perdition model show significant improvement compared to previous methods.

To further enhance the utility of our protocol, we developed a user-friendly web server interface (https://eup.aibtit.com/) that enables efficient, after undergoing comprehensive training on a wide range of diverse samples, EUP webserver achieves end-to-end ubiquitination site prediction across multiple species. It has effectively dismantled the species barrier, thereby enabling highly efficient and accurate prediction of ubiquitination sites spanning species. Moreover, a systematic designed online platform represents a significant step forward in making EUP accessible to a broader scientific community.

## 2. Methods

### 2.1 Datasets

Datasets used in the experiments include both multiple species ubiquitination datasets (S1 Fig). Specifically, data on ubiquitination was obtained from the CPLM 4.0 database (available at https://cplm.biocuckoo.cn/).

The dataset includes 45,902 proteins from multiple species, such as *Arabidopsis thaliana*, *Candida albicans*, *Emericella nidulans*, *Homo sapiens*, *Mus musculus*, *Oryza sativa*, *Rattus norvegicus*, *Saccharomyces cerevisiae*, and *Toxoplasma gondii*. It contains 182,120 experimentally verified ubiquitination sites (labeled ‘1’) and 1,109,668 non-ubiquitination sites (labeled ‘0’). Since the original CPLM 4.0 database lacks protein sequences, the corresponding sequences were obtained from the UniProt database (https://www.uniprot.org).

For model training and evaluation, the dataset was randomly divided into training and test sets in a 7:3 ratio. Additionally, an independent test set comprising 1,191 ubiquitination sites—non-overlapping with the training set—was collected from the GPS-Uber [8] database to further assess the model’s generalization performance.

It is important to note that some modification sites involve non-lysine residues. After reviewing several published tools that use only lysine residues as the reference for ubiquitination prediction, and to control variables and simplify the process [19], we focused exclusively on lysine sites in our prediction model. This includes both ubiquitination and non-ubiquitination sites.

### 2.2 Overview Framework of EUP

**Fig 1:**
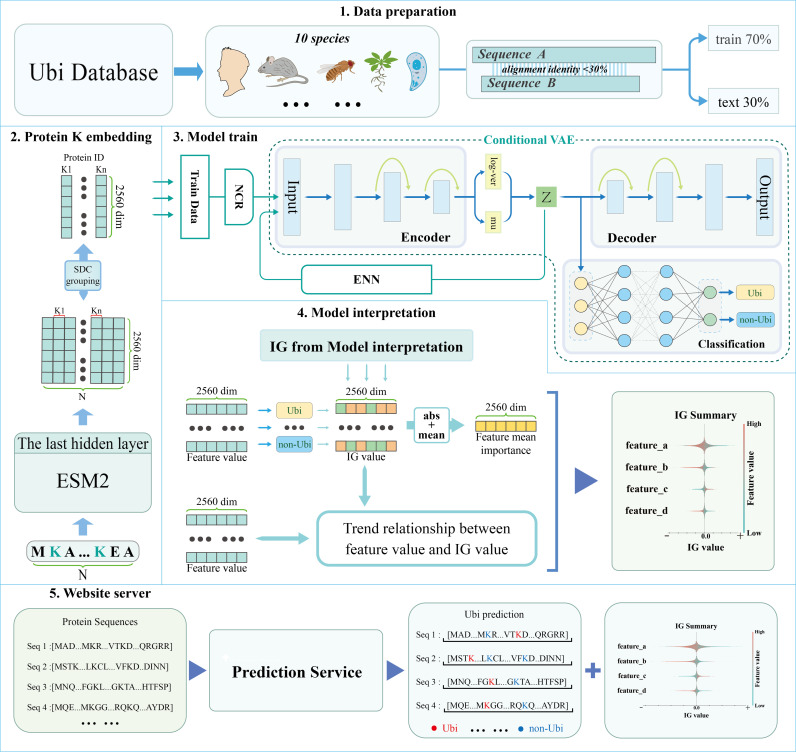
The overview framework of EUP. The diagram delineates the operational process of the EUP website, including: 1. Dara preparation,2.Protein K embedding,3. Model train, 4.Model interpretation, 5.Website server. EUP encompassing the acquisition of protein data from various species. extracting feature embedding of lysine (K) sites based on pretrained ESM2, followed by cVAE for dimensionality reduction of these features to latent feature representation. Then, constructing downstream models based on latent feature representation to predict ubiquitination sites, with creation the interfaceable and interpretable prediction result output.

The framework of EUP (Fig 1) begins with the acquisition of protein data from various species. To extract features from the amino acid sequences, the ESM2 model (version: esm2_t36_3B_UR50D) is employed. Specifically, the feature representation of each lysine residue is extracted from the last hidden layer of ESM2, with each lysine feature having a dimensionality of 2560. These features are then reconstructed and undergo dimensionality reduction using Res-VAE. Since the features are derived by processing the same sequence within a mini-batch, the attention mechanism within the sequence captures the dependencies between them. To further ensure the stability of model training, lysine (K) sites originating from identical sequences are grouped into the same batch. In order to predict ubiquitination sites, four distinct downstream models, driven by Multilayer Perceptron (MLP) and Residue Connection Networks, are deployed to classify each lysine site as either belonging to ubiquitination or not. Additionally, a user-friendly visualization interface is provided on the website, making the model easy to use.

### 2.3 The framework of the conditional-VAE model

This work employs a Conditional Variational Autoencoder (cVAE) model framework that combines the continuous residual Variational Autoencoder (ResVAE) with a classification head for training and inference on the ubiquitination prediction task of lysine (K) sites in protein sequences. ResVAE integrates the idea of residual connections [20] with the Variational Autoencoder (VAE) [21], which has demonstrated strong performance in several biological prediction tasks [22,23]. Using ResVAE, the features of lysine sites (extracted by ESM2) can be efficiently reconstructed, and parameterization techniques in the bottleneck layer help obtain latent vector representations. The latent space is parameterized by a Gaussian distribution, which includes a mean vector (μ) and a log-variance vector (log\_var). Based on this, latent vectors Z are sampled from the standard normal distribution to obtain low-dimensional latent representations corresponding to the original lysine site features:


$$\begin{array}{c} \begin{array}{c} Z=\mu +\varepsilon \cdot \text{exp}\left(\frac{1}{2}\cdot \text{log\textbackslash{}\_var}\right) \end{array} \end{array}$$ (1)

where ε is sampled from a standard normal distribution.

Additionally, the classification head introduces label information while reconstructing the data, effectively constraining the ResVAE to capture features related to lysine ubiquitination. The classification head also serves as a downstream prediction module, which can be directly used for prediction tasks once the cVAE model has been trained.

Therefore, the total loss function of the cVAE model consists of three parts: the reconstruction loss (${ℒ}_{\text{REC}}$, using Root Mean Square Error (RMSE)) based on the prior distribution p and approximate posterior q, the Kullback-Leibler (KL) divergence (${ℒ}_{\text{KLD}}$), and the binary cross-entropy loss (${ℒ}_{\text{CLS}}$) combined with logits. The complete loss function is expressed as:


$$\begin{array}{c} \sigma (x)=\frac{1}{1+e^{-x}} \end{array}$$ (2)

$$\begin{array}{c} {ℒ}_{\text{REC}}=\sqrt{\frac{1}{N\cdot d_{\text{dim}}}\sum_{n=1}^{N}\sum_{i=1}^{d_{\text{dim}}}{\left(y_{\text{rec},n,i}-{\hat{y}}_{\text{rec},n,i}\right)}^{2}} \end{array}$$ (3)

$$\begin{array}{c} {ℒ}_{\text{KLD}}=-\frac{1}{2N}\sum_{n=1}^{N}\sum_{j=1}^{z_{\text{dim}}}\left(1+\text{log}\sigma_{j}^{2(n)}-\mu_{j}^{2(n)}-\sigma_{j}^{2(n)}\right) \end{array}$$ (4)

$$\begin{array}{c} {ℒ}_{\text{CLS}}=-\frac{1}{N}\sum_{n=1}^{N}\left[y_{n}\text{log}\left(\sigma \left({\text{logits}}_{n}\right)\right)+\left(1-y_{n}\right)\text{log}\left(1-\sigma \left({\text{logits}}_{n}\right)\right)\right] \end{array}$$ (5)

$$\begin{array}{c} {ℒ}_{\text{cVAE}}=\left({\alpha \cdot ℒ}_{\text{REC}}+\beta \cdot {ℒ}_{\text{KLD}}\right)+\gamma \cdot {ℒ}_{\text{CLS}} \end{array}$$ (6)

where σ(x) denotes the Sigmoid activation function; N is the number of samples in a batch; $d_{\text{dim}}$ is the feature dimension of each sample in the decoder output; $y_{\text{rec},n,i}$ is the true value of the i-th dimension of the n-th sample (for reconstruction loss); ${\hat{y}}_{\text{rec},n,i}$ is the predicted value of the i-th dimension of the n-th sample; $z_{\text{dim}}$ represents the dimensionality of the latent vector; $\text{log}\sigma_{j}^{2(n)}$ is the log of the variance of the j-th latent variable of the n-th sample (used for numerical stability to avoid zero variance); $\mu_{j}^{2(n)}$ is the squared mean of the j-th latent variable of the n-th sample; $\sigma_{j}^{2(n)}$ is the squared variance of the j-th latent variable of the n-th sample; ${\text{logits}}_{n}$ is the model output for the n-th sample (the predicted value before the Sigmoid activation); $y_{n}$ is the classification label for the n-th sample (0 or 1, used for classification loss); α and β are hyperparameters to balance ${ℒ}_{\text{REC}}$ and ${ℒ}_{\text{KLD}}$, were initially set to 1 in this study. γ is the weight assigned to the classification loss to balance the reconstruction and classification objectives, and was initially set to 1 in this study. Additionally, to evaluate the impact of different weight configurations on model performance, we further tested settings with β set to 0.3. The corresponding results are provided in S1 Table.

Finally, the model architecture and training are implemented using the PyTorch 2.0 framework. The optimizer used is AdamW, with weight decay (L2 regularization) set to 8e-3 and a learning rate of 1e-4. The classification head module is implemented by interchanging the DNNLinearModel and ResDNNmodel as described in the work.

### 2.4 The brief introduce of foundation model

The ESM2 protein language model (3B version), comprising 3 billion parameters and producing 2560-dimensional embeddings, was employed as the primary feature extractor to encode amino acid sequences for downstream prediction. This model was integrated into the core architecture of EUP ubiquitination site prediction model and deployed within the web server. For comparison, we also incorporated ESMc [24] (600M version), a lightweight variant of the ESM 3 family that generates 1152-dimensional embeddings, to evaluate the impact of different model complexity on predictive performance. We also provide the implementation and usage scripts for the ESMc model in our GitHub repository.

### 2.5 Sliding window sequence feature extraction

To compare with traditional window experiments, we performed feature extraction around lysine residues (K). Each individual amino acid residue is encoded into a feature embedding using the ESM2 model. Alterations in the length of the sequence lead to the positions of the K sites, consequently resulting in distinct features for individual K residues. Notably, the extracted K-centered feature is not equivalent to a global pooling of the full amino acid sequence. For each sequence, N amino acids surrounding the lysine site were extracted to form sub-window sequences, which were then used to generate sub-windows features. These sequences were then fed into the ESM2_3B model, generating 2560-dimensional feature vectors.

Additionally, in cases where the sliding window exceeds the boundaries of the protein sequence (i.e., when the lysine site is located near the N- or C-terminus), we apply a direct truncation strategy rather than padding with special symbols. Specifically, the window is truncated to include only the available upstream or downstream residues. This design avoids introducing artificial characters (e.g., ‘X’) that could interfere with the contextual embedding learned by ESM2, ensuring that only authentic biological sequence context contributes to the resulting representation. All sub-window sequences constructed in this way are independently passed through the ESM2 model to obtain lysine-centered, context-specific embeddings.

The mathematical representation for extracting N amino acids centered around a lysine residue can be expressed as:


$$\begin{array}{c} S_{\text{new}}=\left[S_{\text{full}}(i-N),\ldots ,S_{\text{full}}(i-1),S_{\text{full}}(i),S_{\text{full}}(i+1),\ldots ,S_{\text{full}}(i+N)\right] \end{array}$$ (7)

Where $S_{new}\;$represents the new sequence centered on the lysine at position i. $S_{full}$ is the full amino acid sequence. i is the position of the lysine residue in the full sequence. N is the step size of amino acid residues to be extracted as new sequence $S_{new}$, ranging from 5 to 100 and stride is 5. Then $S_{new}$ sequence will be sent to ESM2 for feature extraction.

### 2.6 Data processing and cVAE-based denoise strategy

To address internal sequence homology, which can cause redundancy in the training set and affect model accuracy, CD-hit [25] software was used to remove sequences with more than 30% similarity (Fig 1). After this process, 830,213 tag sites from 26,072 protein sequences across 10 species were retained.

The residual connection module consists of two fully connected layers. The input and output dimensions of the first layer are controlled by two variable parameters, determined by the hyperparameter layer_dims. The input and output dimensions of the second layer are the same and equal to the output dimension of the first layer. A skip connection spans both layers, directly connecting the input of the first layer to the output of the second layer. Additionally, an controller condition is set in this experiment: when the input and output dimensions of the first layer are equalized, an identity mapping is used for the residual connection; otherwise, a linear layer without an activation function is used for the skip connection [20]. The model is constructed based on PyTorch 2.0 and each layer applies batch normalization followed by Leaky ReLU as the activation function.

The loss function for the Res-DNN model is binary cross-entropy, given by the following formula:


$$\begin{array}{c} \text{Binary Cross-Entropy (BCE)}=-\frac{1}{N}\sum_{i=1}^{N}[y_{i}\log\left(p_{i}\right)+\left(1-y_{i}\right)\log\left(1-p_{i}\right)\backslash nonumber \end{array}$$ (8)

Where $p_{i}$ represents the prediction probability of the ubiquitination sites.

Additionally, the Optuna library (version 3.2.0) [26] are deployed to perform a 5-fold stratified cross-validation to search for the optimal hyperparameters of the Res-VAE model, utilizing the AdamW optimizer. The hyperparameters to be optimized include the number of residual connection modules num_blocks, the dimensions of the input and output for each residual connection module layer_dims, the weight decay L2 regularization strength weight_decay, the learning rate lr, a binary parameter as a conditioner to trigger adding loss weights to the minority class (ubiquitination positive labels), and the number of training epochs.

The optimization objective is to maximize the MCC (Matthews Correlation Coefficient) value on the validation set. The Optuna algorithm’s default TPE (Tree-structured Parzen Estimator) is used to search for potential optimal hyperparameters, and the default pruning strategy is employed.

In this experiment, we adopted a denoising strategy based on a conditional variational autoencoder (cVAE) for data cleaning and noise reduction. This strategy first employs the Neighborhood Cleaning Rule (NCR) [27] (implemented via the imbalanced-learn toolkit) to remove majority-class labeled data identified as noise. The core mechanism involves eliminating majority-class instances misclassified by their nearest neighbors, thereby achieving data balance and noise reduction. Subsequently, the cVAE architecture, fitted on the denoised data, is used to obtain low-dimensional Z vectors. These vectors, combined with label data, are further processed using Edited Nearest Neighbours (ENN) [27] to eliminate additional noisy samples, thereby enhancing data quality and improving model generalization performance.

### 2.7 Evaluation matrix of model prediction

The predictive performance of the classification models across different test datasets is evaluated using several assessment metrics, including precision, recall, F1 score, accuracy (ACC), and the Matthews correlation coefficient (MCC) [28]. Initially, these metrics were calculated at a fixed Decision Threshold (DT) of 0.4. To ensure fair and consistent comparisons of these metrics values across models, we have additionally evaluated model performance at a fixed false positive rate (FPR) of 5%. The formulas for these calculations are provided below.


$$\begin{array}{c} \begin{array}{cc} \text{precision}= & \;\frac{\text{TP}}{\text{TP}+\text{FP}},\\ {}[8pt]\text{accuracy}= & \;\frac{\text{TP}+\text{TN}}{\text{TP}+\text{TN}+\text{FP}+\text{FN}},\\ {}[8pt]\text{recall }= & \;\frac{\text{TP}}{\text{FP}+\text{FN}},\\ {}[8pt]F1_{\text{score}}\;= & \;\frac{\text{precision}\times \text{recall}}{\text{precision}+\text{recall}},\\ {}[8pt]\text{MCC }= & \;\frac{\left(\text{TP}\times \text{TN}\right)-\left(\text{FP}\times \text{FN}\right)}{\sqrt{\left(\text{TP}+\text{FP}\right)\times \left(\text{TP}+\text{FN}\right)\times \left(\text{TN}+\text{FP}\right)\times \left(\text{TN}+\text{FN}\right)}}. \end{array} \end{array}$$ (9)

Where ubiquitination-positive sites are considered as the positive class, TP denotes true positives, FP denotes false positives, FN denotes false negatives, and TN denotes true negatives.

Additionally, the model’s performance at various positive thresholds is evaluated by calculating the area under the receiver operating characteristic curve (AUC) and the precision-recall curve. The AUC curve involves calculating the true positive rate (TPR) and false positive rate (FPR) for different threshold settings, while the precision-recall curve involves calculating precision and recall for different thresholds. The implementation of the aforementioned algorithms is based on the sklearn library (version 0.24.2.).

### 2.8 Feature importance and contribution evaluation methods

To interpret the model’s predictions, we employed SHAP for feature importance visualization [29], applied to the 2560-dimensional feature vectors representing ubiquitination sites. First, we adopt the Integrated Gradients (IG) [30] to calculate each feature attribution value. This is based on determining the gradient of the model’s output with respect to each feature, compare to baseline. Then, feature attribution values were visualized using SHAP package (version: 0.47.2) to identify the contribution mode of each feature to the model’s decision-making process [31].

The core idea of the IG is to compute the input feature’s marginal contributions via a path integral along the straight-line path between a baseline input and the actual data input. The Integrated Gradients (IG) for a feature $x_{i}$ are computed as:


$$\begin{array}{c} {\text{IG}}_{i}=\left(x_{i}{-x}_{i}^{\text{baseline}}\right)\cdot \int_{\alpha =0}^{1}\frac{\partial F\left(x^{\text{baseline}}+\alpha \cdot \left(x{-x}^{\text{baseline}}\right)\right)}{\partial x_{i}}\;d\alpha \end{array}$$ (10)

Where x represents the target input (e.g., `X_train_tensor`). While $x^{\text{baseline}}$ refers to the baseline input, which is typically a tensor filled with all zeros.

### 2.9 Web server construction method

EUP website is constructed based on the Flask framework (version: 3.0.2), Notably, we set Flask decorators with predefined routes, mapping URLs (Uniform Resource Locator: Used on the Internet to identify the address of a resource) to corresponding backend definitions. Jinja2 (version:3.1.3) is employed as the template engine for the frontend, facilitating the creation of dynamic HTML content. JavaScript is then applied to enrich the user interface and enhance interactivity.

Flask backend is used to construct the website API, ensuring seamless communication between the frontend and backend. The design incorporates considerations for security and performance, with robust error handling and logging mechanisms. The application is containerized using Docker (version:27.3.0) to facilitate deployment and ensure consistency across different environments. It is ultimately hosted on a cloud server to provide a seamless user interaction experience.

For the predicting ubiquitination sites. Users can submit their protein sequences in FASTA format, either by direct input or by uploading a FASTA file. Upon submission, the system processes the sequences to identify potential ubiquitination sites. Additionally, the EUP system generates a comprehensive set of 2560-dimensional features associated with each predicted site. These features encapsulate various sequence and structural attributes relevant to the ubiquitination process. The output, including the ubiquitination sites and the corresponding high-dimensional feature data, is available for download. Users can further input the prediction outcome data to interpretable analysis module for feature attribution analysis, thereby obtaining the contribution mode of different features. It offers researchers valuable insights for further analysis in protein function studies and drug development endeavors.

This methodology presents an efficient approach to ubiquitination site prediction, equipping researchers with a rich dataset that can be instrumental in advancing understanding in the fields of proteomics and therapeutics.

## 3. Results

### 3.1 Evaluation of EUP model Ubi-prediction performance

**Fig 2:**
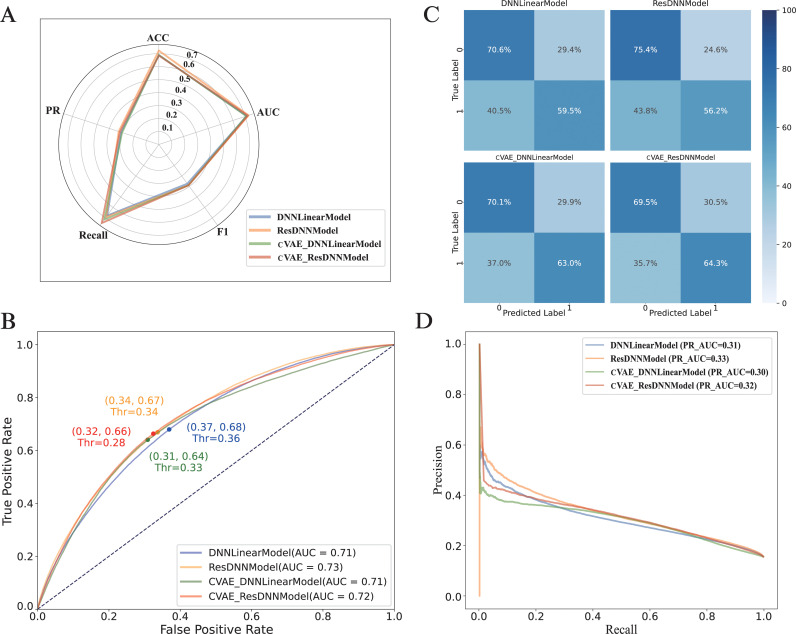
The accuracy outcomes of ubiquitination site predictions for multiple species, encompassing. (a) Radar chart depicting the precision of predictions, (b) AUROC curve graph for assessing, (c) a confusion matrix for predictive analysis predictive performance, and (d) AUPRC curve graph for a comprehensive evaluation of prediction accuracy.

In this study, four models with multiple architectures are evaluated to identify the optimal downstream model deployed in EUP, including DNNLinearModel, ResDNNModel, cVAE_DNNLinearModel, and cVAE_ResDNNModel (Table 1), This section presents results based on an integrated training dataset comprising 10 species, with a 30% homology reduction threshold applied to training one integrated model. Inference was performed in a model prediction DT of 0.4 was applied across all data in a species-agnostic manner. A rigorous 5-fold cross-validation experiment was performed, and the optimized model was selected based on a set of metrics, including Accuracy, F1-Score, Recall, Matthew’s Correlation Coefficient (MCC), Area Under the Receiver Operating Characteristic Curve (AUROC), and Area Under the Precision-Recall Curve (AUPRC) (Fig 2A). The DNNLinear Model, established as a baseline, exhibited an Accuracy of 0.689 and an AUROC of 0.712; however, it recorded lower F1-Score, Recall, and MCC values of 0.373, 0.595, and 0.231, respectively. Additionally, compared to DNNLinearModel, the ResDNNModel, despite its increased complexity, showed no significant improvement in predictive performance. This suggests that the feature extraction from the last hidden layer of ESM2 (3B) may already provide a sufficiently complex and abstract representation, rendering the additional model complexity unnecessary.

**Table 1:** Evaluation of Predictive Performance Across Models.

Model Name	MCC	F1_Score	Recall	Accuracy	AUC	PR
ResDNN	0.251	0.389	0.562	0.724	0.727	0.325
DNNLiner	0.231	0.374	0.595	0.689	0.712	0.306
cVAEResDNN	0.255	0.390	0.643	0.686	0.722	0.311
cVAEDNNLiner	0.254	0.389	0.633	0.691	0.708	0.298

In comparison, the cVAE_DNNLinearModel, which employs a conditional Variational Autoencoder (cVAE) to reduce the feature dimension from 2560 to 100 while incorporating additional noise reduction through NCR methods, demonstrated improved performance, achieving an Accuracy of 0.691 and an AUROC of 0.708. The F1-Score for this model increased to 0.389, respectively, with the MCC reaching 0.254, indicating a substantial enhancement in the model’s predictive capabilities. The cVAE_ResDNNModel, utilizing the same cVAE and NCR design, achieved the highest Accuracy of 0.686 and an AUROC of 0.722, with F1-Score and Recall values of 0.390 and 0.643, respectively. The MCC for this model was 0.255, further validating its superior classification quality.

A comparative analysis across all evaluation metrics demonstrated the superiority of the cVAE_ResDNNModel over the baseline and other models, underscoring the significant role of NCR in enhancing the model’s performance by addressing issues related to imbalanced and low-quality datasets. Additionally, the integration of cVAE not only refines feature representation but also improves model inference efficiency, which is crucial for real-time applications. However, the feature representation in the ESM2 model, which requires information to be fed forward through the entire architecture, was identified as a bottleneck in inference efficiency. Future research directions may involve model distillation or layer reduction of ESM2 to achieve higher computational efficiency without compromising predictive performance. The Receiver Operating Characteristic (ROC) curves (Fig 2B and 2C) illustrated the diagnostic performance of the models, demonstrating the predictive balance achieved.

The integration of the Conditional Variational Autoencoder (cVAE) into the DNNLinear Model and ResDNN Model has resulted in notable improvements in the accuracy of multi-species ubiquitination site predictions. Specifically, the ResDNNModel achieved an AUC of 0.727, while the DNNLinearModel recorded an AUC of 0.711. When enhanced with cVAE, the cVAE_Res_DNNModel and cVAE_DNNLinear Model reached AUCs of 0.722 and 0.708, respectively. While the increase in AUC values was modest, the integration of cVAE significantly enhanced the true positive rates: the cVAE_DNNLinear Model showed an improvement of approximately 5.88% over the DNNLinear Model, and the cVAE_ResDNN Model improved by about 14.41% compared to the ResDNN Model. These improvements underscore the cVAE models’ enhanced balance between sensitivity and specificity, demonstrating their capacity to more effectively discern complex patterns in the data. Moreover, the adoption of techniques such as NCR and ENN havehas further increased the robustness and reliability of these models, contributing to their overall performance enhancements.

Furthermore, the Precision-Recall (PR) curves (Fig 2D) provide additional insights into the ROC analysis, which are particularly valuable for imbalanced datasets. The DNNLinear Model recorded a PRAUC of 0.30, while the ResDNN Model demonstrated a slightly higher PRAUC of 0.33. Integration of the cVAE into these models resulted in a PRAUC of 0.30 for the cVAE_DNNLinear Model and 0.32 for the cVAE_ResDNN Model. Although the inclusion of cVAE did not markedly increase the average PRAUC values, it suggested improvements in the balance between precision and recall. These findings highlight the benefits of utilizing cVAE in managing complex and imbalanced datasets, particularly in terms of enhancing model generalizability and robustness.

Further analysis of the PR curves shows that the cVAE-integrated models maintained higher precision at specified recall levels, indicating their effectiveness in minimizing false positives. This supports the utility of cVAE integration for refining feature representations and improving the precision-recall balance.

In conclusion, incorporating cVAE into predictive models has significantly enhanced their capability to process imbalanced and low-quality datasets, refine feature representations, and boost inference efficiency. These improvements position these models as strong contenders for achieving high-accuracy predictions.

To investigate how different thresholding strategies affect classification performance, all models were evaluated under a fixed (FPR = 5%) and compared against the baseline condition using a fixed (DT = 0.4).The comparative results are summarized in S2 Table. We observed that recall, F1-score, and MCC were consistently higher across all models (DNNLinearModel, ResDNNModel, cVAE_DNNLinearModel, and cVAE_ResDNNModel) when evaluated under a fixed decision threshold (DT) of 0.4. For instance, the cVAE_ResDNNModel achieved a recall of 0.643, an F1-score of 0.390, and an MCC of 0.255 under DT = 0.4, whereas under a fixed false positive rate (FPR) of 5%, the same model’s recall dropped to 0.172, F1-score to 0.239, and MCC to 0.175. A similar pattern was observed for the DNNLinear model, with recall decreasing from 0.595 to 0.175, and MCC from 0.231 to 0.179. In contrast, accuracy was uniformly higher under FPR = 5%, with all models achieving values above 0.826. For instance, the accuracy of the cVAE_DNNLinear Model increased from 0.691 (DT = 0.4) to 0.826 (FPR = 5%). As expected, AUC and PR-AUC remained unchanged across both thresholding strategies for each model since these are threshold-independent metrics. Accuracy improved under a stringent FPR threshold (5%), However this does not necessarily indicate optimal model performance, particularly given that accuracy is not a robust metric for evaluating imbalanced datasets. Considering all evaluation metrics, this indicates that the model performs suboptimal under a strict low-error setting (FPR = 5%). Accordingly, we selected the default decision threshold (DT = 0.4) for deployment on the web server.

In addition, to evaluate the statistical significance of the differences in ROC AUROC values among the four models (DNNLinearModel, ResDNNModel, cVAE_DNNLinearModel, and cVAE_ResDNNModel), and to rule out the possibility that these differences are due to random error, we conducted statistical tests [32] to determine whether the observed performance differences were significant (Fig 2B). pairwise DeLong tests were conducted across all model comparisons (S3 Table). The results revealed statistically significant differences in AUC values (p < 0.05) across all model. For example, the AUC difference between the DNNLinear model and the cVAE_ResDNN model was 0.0102 (p = 4.49 × 10 ⁻ ¹³), and the difference between the ResDNN model and the cVAE_ResDNN model was 0.0051 (p = 7.14 × 10 ⁻ ⁵). These findings suggest that the modest increases in AUC scores achieved by the cVAE-integrated models are statistically significant. This phenomenon suggests that the EUP framework based on a pretrained large model is relatively insensitive to the architecture of downstream classifiers (at least under the current experimental setting). Although the integration of the NCR into the cVAE model leads to a slight but statistically significant performance improvement, the final performance differences remain marginal. These findings further support the rationale of developing task-specific models upon pretrained foundation models.

### 3.2 Model inference latency analysis

To provide a more comprehensive comparison of the practicality of our method, an evaluation experiment of inference time is conducted across different models. Since inference efficiency is primarily determined by the model architecture rather than the size of the protein dataset, we randomly selected 32 proteins within the 450–550 bp range from the testing set, resulting in a total of 1,163 ubiquitination prediction sites. The average length of each protein sequence is 515.37 bp, and on average, each protein sequence harbors 36.37 ubiquitination sites are pending prediction. The results revealed that cVAE base inference model significantly reduced computational latency (Table 2).

**Table 2:** Inference latency analysis for different models.

Model	Batch_size	Test_time
ResDNN	1	0.267
ResDNN	32	0.228
ResDNN	64	0.186
DNNLiner	1	0.212
DNNLiner	32	0.193
DNNLiner	64	0.179
cVAEResDNN	1	0.245
cVAEResDNN	32	0.216
cVAEResDNN	64	0.189
cVAEDNNLiner	1	0.210
cVAEDNNLiner	32	0.200
cVAEDNNLiner	64	0.186

Specifically, the DNNLinear Model exhibited a test time of 0.212 seconds for one amino acid sequence as input (batch size of 1), which was reduced to 0.179 seconds when input number of sequence is set to 64 (batch size of 64). In comparison, the cVAEDNNLiner Model exhibited a test time of 0.210 seconds for a batch size of 1, and 0.186 seconds for a batch size of 64. Overall, the latter showed a substantial decrease in time consumption by 15.6% and 11.4%, respectively.

Similarly, the ResDNN Model exhibited a test time of 0.267 seconds for a batch size of 1, which was reduced to 0.186 seconds for a batch size of 64. The cVAEResDNN Model exhibited a test time of 0.245 seconds for a batch size of 1, and 0.189 seconds for a batch size of 64, reflecting improvements of 30.3% and 22.6%, respectively.

These results highlighted the significant advantages of incorporating cVAEs into downstream model inference, particularly in high-throughput applications where fast inference was essential. Although the integration of cVAE led to a slight increase in computation time with a batch size of 1, the overall reduction in computational latency ensured that more complex models leveraging cVAE maintained competitive inference speeds. This balance between increased model complexity and efficient processing time underscored the potential of cVAE to enhance model performance, making it a compelling choice for real-time applications. The findings suggested that the integration of cVAE into downstream models was an effective strategy for optimizing both inference speed and accuracy, particularly in scenarios requiring high-throughput processing.

### 3.3 Model predication evaluation for sub-windows feature

**Fig 3:**
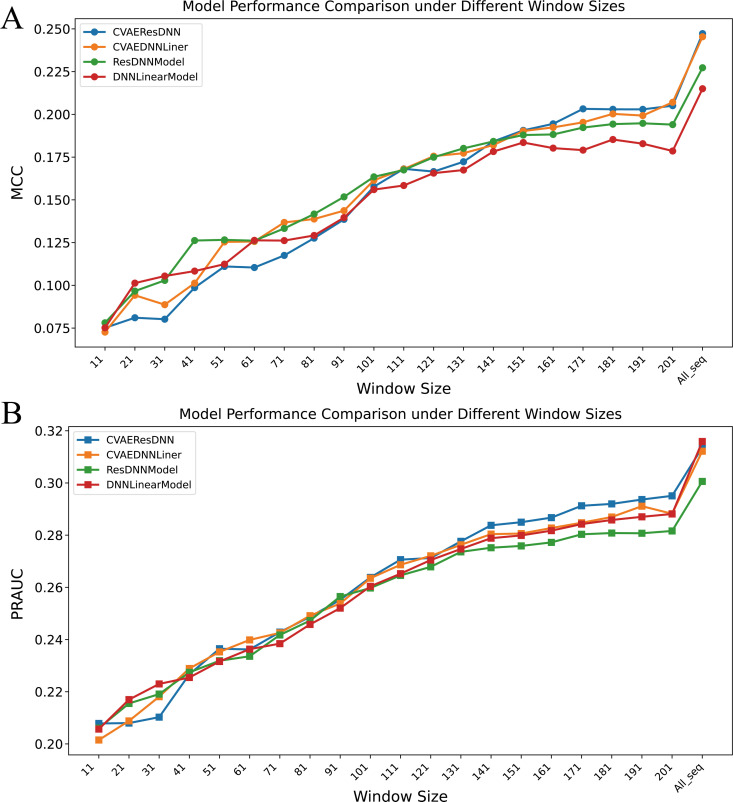
Model performance under different window sequences. (A) Performance measured by MCC (Matthews Correlation Coefficient).(B) Performance measured by PRAUC (Precision-Recall Area Under the Curve).The “window size” on the x-axis indicates the length of the local sequence centered on the lysine (K) residue, constructed by expanding 5×n amino acids forward and backward from the lysine site center(2×5×n+1, where $n\in {\mathbb{Z}}^{+}$ and n≥1). These sequence windows were encoded using ESM2 to extract context-specific features for model input. The value “All_seq” corresponds to the full-length protein sequence.

For a more comprehensive evaluation of feature extraction methods effectiveness, we tested four models’ prediction performance using feature extraction from lysine site based on full sequences with those based on subsequences. The test data were drawn from 3000 proteins randomly selected within the 450–550 bp range from the testing set, totaling 23827 ubiquitination prediction sites. Performance was assessed using MCC and PR-AUC metrics (Fig 3A and 3B).

Specifically, when excluding the full sequence, the cVAE_DNNLinear model achieved the highest MCC value of 0.238 at a window size of 201, while the cVAE_ResDNN model reached 0.223 at the same window size (Fig 3A). These values, although lower than full-sequence performance, still outperformed their counterparts at smaller windows, highlighting the advantage of long-range context. In contrast, the DNNLinear model showed a gradual improvement in MCC from 0.006 to 0.286, and the ResDNN model increased from 0.080 to 0.477 as the window size increased from 11 to full sequence. However, neither non-cVAE model surpassed the performance of cVAE-based models at any window size, suggesting that cVAEs contribute significantly to modeling long-range dependencies and enhancing feature representations.

This observation supports the idea that cVAE-integrated models can maintain robust performance even with limited input context, and that incorporating long-range sequence information allows cVAE to reconstruct and enrich the latent features extracted from the protein language model. The performance trends observed in PR-AUC scores (Fig 3B) were consistent with MCC findings, further confirming this advantage.

Moreover, the performance of all models improved with sub window extend to full sequence, underscoring the more context for in extracting single site corresponding feature from large language model ESM2, the more performance improved in downstream model. In comparison, a classic non-pretrained small model has strict limitations on window partitioning in windows, and although smaller windows can lead to faster model inference speed, extending the background information of the window cannot effectively improve the model’s performance. This finding also supports that while window methods can capture local features, global information is crucial for modeling the complete characteristics of the sequence and enhancing predictive accuracy. Therefore, combining local window methods with global information may be an effective strategy to improve model performance, especially when utilizing cVAE to further enhance feature embedding with reconstruct learning.

### 3.4 Comparison of different foundation models and independent dataset validation

To further assess the impact of protein feature representations derived from different foundation models, we performed an ablation analysis comparing ESM2 and ESMc embeddings across four predictive architectures—ResDNN, DNNLinear, cVAE_ResDNN, and cVAE_DNNLinear—under three denoising strategies: None, NCR, and NCR-ENN (NCRENN) (S4 Table). Overall, models utilizing ESM2 embeddings consistently outperformed their ESMc-based counterparts in terms of both MCC and F1-score, particularly when combined with NCR-based denoising. For example, the cVAE_DNNLinear model with ESM2 + NCR achieved the highest performance (MCC = 0.254, F1 = 0.389), surpassing both ESM2 + NCRENN (MCC = 0.246, F1 = 0.382) and ESMc+NCRENN (MCC = 0.232, F1 = 0.373) configurations. A similar trend was observed in the cVAE_ResDNN model, where ESM2 + NCR attained MCC = 0.255 and F1 = 0.390, outperforming ESMc + NCR (MCC = 0.246, F1 = 0.383). Notably, NCRENN demonstrated greater enhancements in recall than in MCC in several instances (e.g., Recall = 0.771 for cVAE_ResDNN with ESM2 + NCRENN vs. 0.643 with NCR), suggesting its utility in improving model sensitivity.

Building upon these results, we further evaluated the generalization ability of all configurations on an independent validation set (S5 Table). Consistently, models incorporating ESM2 embeddings outperformed those using ESMc, with cVAE_DNNLinear + ESM2 + NCR exhibiting the strongest generalization (MCC = 0.231, F1 = 0.660, Recall = 0.757, Accuracy = 0.610). Other models, such as ResDNN and DNNLinear, demonstrated inferior performance across all feature-denoising combinations. Although ESMc + NCR occasionally resulted in high recall (e.g., 0.755 for ResDNN), the corresponding decreases in MCC and F1-score highlighted a trade-off in predictive balance. Importantly, both NCR and NCRENN consistently contributed to performance improvements, underscoring the robustness conferred by denoising strategies.

Collectively, these results demonstrate that the integration of a conditional variational autoencoder (cVAE) architecture, ESM2-derived embeddings, and NCR-based denoising—particularly NCR-ENN—constitutes a robust and generalizable framework for ubiquitination site prediction. This approach is particularly well-suited for real-world deployment in scenarios involving unseen or taxonomically diverse protein sequences, where both predictive generalization and sensitivity are essential.

### 3.5 Multi-species model performance evaluation

**Fig 4:**
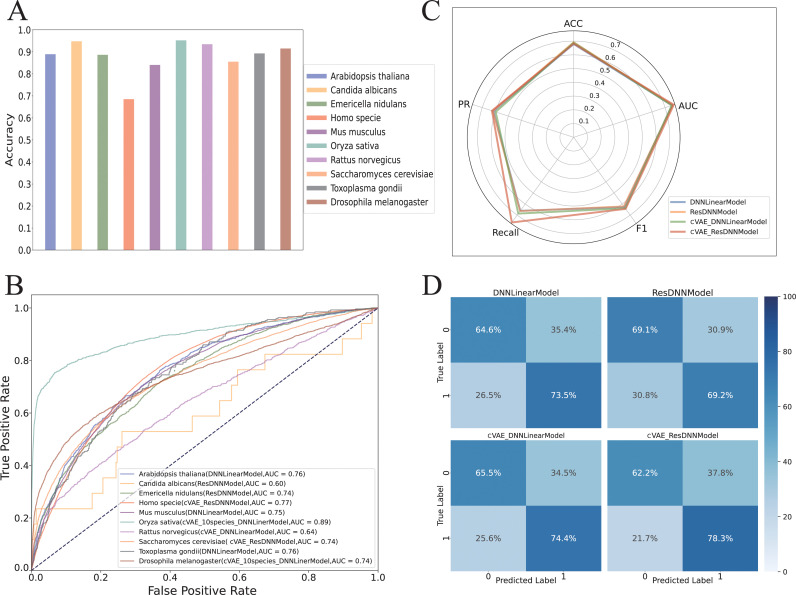
The accuracy outcomes of ubiquitination site predictions for multiple species, encompassing. (a) A bar chart illustrating the accuracy (ACC) of the best-performing models for ubiquitination site prediction across ten species, highlighting model variations and species-specific performance differences.(b) A receiver operating characteristic (ROC) curve for the optimal models across ten species, with area under the curve (AUC) values reflecting classification performance in ubiquitination site prediction.(c) A radar chart displaying a comprehensive performance comparison of four models for Homo sapiens, showcasing balanced performance across all models.(d) Confusion matrices of the four models applied to Homo sapiens, visualizing true positive and negative rates, as well as misclassification rates in a heatmap format.

To account for the substantial variations in model prediction performance among species, we performed evaluation and distinct analyses for each species using their optimal model weights including DNNLinearModel, ResDNNModel, cVAE_100_DNNLinearModel, cVAE_100_ResDNNModel and multi-species Model (S6 Table). Specifically, for each species, we selected the best-performing model among the five trained architectures based on validation performance, and used its prediction accuracy (ACC) as the representative evaluation value (Fig 4A), we observed an ACC range of 0.682 to 0.945 across 10 species, with *Oryza sativa* exhibiting the highest ACC at 0.945. Conversely, *Homo sapiens* recorded the lowest ACC of 0.682. This variation might stem from the substantial evolutionary divergence in protein amino acid sequences between the fungal species *Oryza sativa* and the *Homo sapiens*. Moreover, the significantly larger and imbalanced dataset for *Homo sapiens* might have influenced this result. Moreover, we plotted the AUC (Area Under the Curve) for all species (Fig 4B), which provided a clearer assessment of the model’s prediction performance in different threshold and facilitate more intuitively comparison between species. The AUC values varied from 0.597 to 0.895. Notably, despite its high ACC, *Candida albicans* had a less optimal AUC. In contrast, *Homo sapiens*, with a more balanced dataset, achieved an AUC of 0.767, ranking it third and compensating for the lower ACC.

These findings suggest that while the models generally performed comparably across species, certain evolutionary or data-specific factors might lead to meaningful divergence in predictive capabilities.

Additionally, *Oryza sativa* demonstrated AUC values of 0.895, respectively, with corresponding ACC values above 0.9, indicating a strong fit to the multi-species Model prediction.

In evaluating model selection across various species, distinct patterns have emerged, showcasing optimal model performance that often deviates from single-species training approaches. For example, *Oryza sativa* demonstrated superior results with the multi-species model labeled as ‘10_species’, which likely benefits from the high homology between its amino acid sequences and those found in other species within the dataset. This suggests that shared characteristics across species can enhance model performance due to more generalized learning.

Conversely, for species such as *Arabidopsis thaliana* and *Toxoplasma gondii*, simpler models like the DNNLinear Model were more effective than the more complex cVAE frameworks, indicating a potential mismatch between model complexity and dataset characteristics. This divergence suggests that the inherent simplicity of some datasets may be better captured by less complex models, which could more directly represent the underlying patterns without overfitting to noise. Additionally, species-specific customizations have led to tailored models such as the cVAE_ResDNN Model for *Homo sapiens* (tagged as ‘Homo_NCR’), highlighting the need for specialized approaches in datasets with unique features (Fig 4C and 4D). The use of NCR (Noise-Contrastive Regularization) in this context emphasizes the adaptation to specific data challenges, enhancing the model’s ability to discern relevant biological signals from noise.

### 3.6 Key feature identification across multiple species

**Fig 5:**
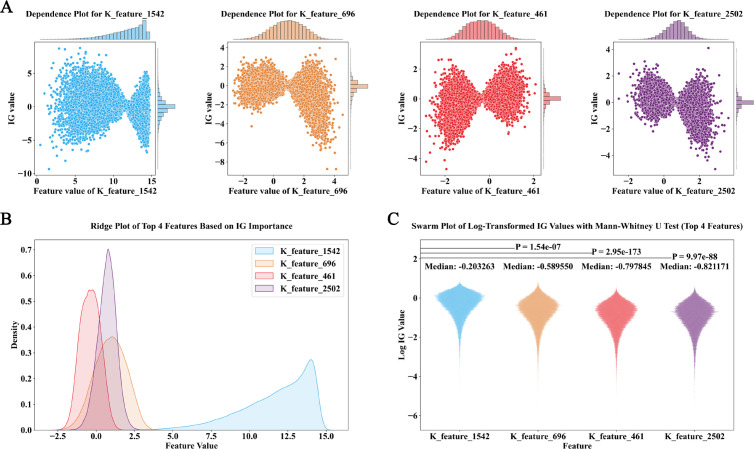
Integrated Gradients (IG) analysis for ubiquitination site predictions in Homo sapiens, encompassing. (a) Feature dependence plots for the top four most important features (K_feature_1542, K_feature_696, K_feature_461, and K_feature_2502), illustrating the relationship between feature values and feature attribution (IG) values..(b) A dense plot displaying the density distribution of the top four features’ values, highlighting variations in feature value distributions across K_feature_1542, K_feature_696, K_feature_461, and K_feature_2502.(c) A swarm plot of log-transformed IG values for the top four features, accompanied by Mann-Whitney U tests for statistical significance. The median log-transformed IG values and p-values indicate distinct patterns in feature importance, with K_feature_1542 showing the highest impact.

In the analysis of prediction patterns from models of ten species and the selection of key features for each species, we used the captum package [33] on the EUP online server to calculate feature importance (IG values) based on the model. Additionally, we utilized the SHAP package to visualize the contribution patterns of different features across species, which helps in understanding the decision-making process of the models in different species (Fig 5).

Among these ten species, the feature importance plot (S2 Fig) displays the top 20 features and their attribution patterns as identified by the model. Among these features, K_feature_1542 consistently ranks first in all species, indicating its crucial role in predicting whether a lysine site is ubiquitinated. Furthermore, there is a negative correlation between the feature value of K_feature_1542 and its IG value, meaning that as the feature value increases, the IG value shifts from positive to negative. This suggests that as the feature strengthens, it negatively impacts the model’s prediction, making it more likely to predict the site as non-ubiquitinated.

In animal species including *Drosophila melanogaster*, *Homo sapiens*, *Mus musculusand*, and *Rattus norvegicus* (S3 Fig), the contributions of K_feature_696, K_featuere_461, and K_feature_1512 remain high and stable, indicating that these features significantly influence the model’s prediction of whether a lysine site is ubiquitinated. Notably, in *Drosophila melanogaster*, aside from K_feature_1542, the contribution rankings of other features fluctuate considerably compared to *Homo sapiens, Mus musculusand*, and *Rattus norvegicus*. Moreover, K_feature_696 does not exhibit a clear positive-negative correlation, which may be related to significant species differences between *Drosophila melanogaster* and the other three animal species.

In plant species including *Arabidopsis thaliana* and *Oryza sativa* (S4 Fig), K_feature_696 and K_feature_461 maintain high contribution rankings. However, in *Oryza sativa*, K_feature_1512 and K_feature_1904, two features with a clear positive-negative correlation between their feature values and IG values, rank third and fourth, respectively. Additionally, the positive correlation between the feature value and IG value for K_696 in *Oryza sativa* is more pronounced compared to *Arabidopsis thaliana*, highlighting the stronger association between K_feature_696, K_feature_1512, and K_feature_1904 with ubiquitination site prediction in *Oryza sativa*. This may help explain why *Oryza sativa* has a higher overall prediction score than *Arabidopsis thaliana*.

Similarly, in microbial species including *Emericella nidulans*, *Candida albicans*, *Saccharomyces cerevisiae*, and *Toxoplasma gondii* (S5 Fig), except for *Toxoplasma gondii*, the contribution ranking of K_feature_696 remains stable in second place. In *Toxoplasma gondii* (a protozoan), K_feature_461 ranks second. Notably, there are significant differences in the contribution rankings of high-ranking features across the microbial species, which may be due to differences in phylogenetic relationships between the species. The evolutionary divergence of protein structures has affected the feature importance rankings. Moreover, in *Candida albicans*, K_feature_461 is no longer present among the top 20 features, and its prediction score is significantly lower compared to the other three microbial species. This could be related to the smaller training dataset for *Candida albicans*, making the sample size more influential in feature importance calculations.

Overall, our experiments provide valuable insights into key features and their contribution patterns across ten species. While these results highlight consistent patterns of feature attribution across species, it is important to note that the features representations extracted from the ESM2 model are not directly reflect biological functions or elements such as sequence motifs and functional domains. Therefore, feature attribution methods, such as SHAP and Integrated Gradients, are designed to help interpret how input features contribute to the EUP model’s predictions and should not be simply interpreted as reflecting biological meaning.

To enable a more comprehensive comparison of model interpretability and to understand the differences among various interpretability algorithms, we additionally employed LIME [34] (Local Interpretable Model-Agnostic Explanations). To assess the consistency across interpretability methods, we compared feature importance scores derived from LIME and SHAP for all ten species. The comparison results are presented as scatter plots (S6 Fig) and Spearman rank correlation heatmaps (S7 Fig).

In S6 Fig, the scatter plots demonstrate a strong positive linear relationship between LIME and SHAP importance scores across most species. For example, in *Homo sapiens, Mus musculus, Oryza sativa, and Drosophila melanogaster*, the data points cluster closely along the diagonal, indicating highly consistent feature rankings between the two methods. Only minor deviations are observed. In species with smaller or more imbalanced datasets, such as *Candida albicans*, the correlation remains moderately strong, though the feature distributions show slightly more dispersion. These observations are corroborated by the high Spearman correlation coefficients shown in S7 Fig, which range from 0.96 to 0.98 (p < 0.01) in most species. Taken together, the statistical evidence affirms the robustness and reliability of the model’s feature attribution across diverse interpretability frameworks, reinforcing confidence in the stability of learned feature attributions across methods and species. As such, the discussion of biological meaning should be interpreted cautiously and regarded as suggestive rather than conclusive. Features like K_feature_1542, which show species-specific variations, may still reflect underlying biological patterns, potentially associated with differences in ubiquitination systems and species-specific regulatory requirements [35]. While some features remain consistent across species, they also highlight the diversity of ubiquitination mechanisms, which have evolved to meet the specific needs of different organisms. Understanding the differences between these shared and unique features will provide inspiration for future feature representation studies and enhance our understanding of the ubiquitination system and its role in biological regulation and disease development.

### 3.7 EUP online web server

Based on a comprehensive evaluation of various models, the high-performing cVAE_ResDNNModel, which integrates cVAE with NCR_ENN, has demonstrated exceptional predictive accuracy across multiple species and has been seamlessly incorporated into the EUP platform. This model is now a key component of an interactive interface on the EUP website, enabling users to conduct online analyses by uploading FASTA files to generate predictions for ubiquitination sites. The results are meticulously organized in an Excel file, where each column is clearly labeled with corresponding protein names, sequence meta-information, length, lysine sites, and ubiquitination prediction labels. Furthermore, the output includes a sophisticated ESM2 2560-dimensional embedding feature for each lysine site, designed to facilitate advanced research and experimental investigations.

Additionally, the EUP platform enhances the transparency of its predictive processes by supporting high-throughput calculations for feature attribution. This functionality allows for the detailed statistical analysis and visualization of feature attribution values, aiding in the elucidation of the mechanisms through which key features contribute to the prediction of ubiquitination sites. Moreover, since the prediction performance for certain species may not be optimal under the cross-species model, we have made species-specific models available on our GitHub repository (https://github.com/EUP-laboratory/ESM2-Ubiquitination-Prediction/tree/main/best_model_in_speciedata). These models are provided to support more accurate inference and offer high-confidence predictions tailored to individual species, thereby facilitating further research within the academic community.

## 4. Discussion

Based on large-scale foundation model, we construct EUP for predicting ubiquitination with a balance of speed and accuracy. The core model cVAE dimension and reconstruct feature embedding are proved to significantly enhanced the model’s evaluation accuracy by reducing unnecessary noise, while also balance runtime speed. By integrating the feature attribution algorithm, EUP is capable of leveraging the intricacies within the key features of ubiquitination prediction across different species, understanding their contribution patterns, thereby enhancing the model interpretation and paving the way for future research. Furthermore, EUP is made available as a user-friendly web server, facilitating the prediction of ubiquitination sites within protein sequences and enhancing accessibility for researchers across different domains.

Notably, the shared features identified by EUP suggest their potential involvement in the ubiquitination process, indicating the capture of evolutionarily conserved traits. At the same time, the differential importance of certain features across species highlights inter-species variability in the ubiquitination mechanism. Afterall, these features are derived from ESM2’s rich representation and abstraction of amino acid sequences, demonstrating the model’s ability to leverage evolutionary information.

Additionally, it is important to note that SHAP and other feature attribution techniques integrated with EUP cannot directly reveal biological information related to ubiquitination or evolutionary signals encoded in the features. Although prior studies [16,24] have shown that the representations learned by ESM-2 can capture protein ubiquitination and evolutionary information, the intrinsic complexity of ESM-2 and the compressed nature of its encoded features [36] make it difficult to extract such biological insights directly through attribution methods like SHAP. The current implementation of EUP focuses on ranking feature importance and analyzing cross-species similarities or differences, which can provide interpretability for understanding how the EUP ubiquitination prediction model adapts to interspecies variability. Future work will require the integration of feature decompression techniques, such as Semantic Autoencoders (SAEs) [36], to disentangle and encode the representations into interpretable semantic units. This will be essential for gaining deeper insights into the biological knowledge implicitly embedded in large model embeddings.

As a complement to PLM (protein language model) research [37], this study demonstrates that large protein language models can effectively perform prediction tasks at the single token (amino acid) level without domain knowledge explicitly required. This effectiveness may be driven by PLMs embedding vast amounts of information from protein sequences during pretraining [16,38,39]. Furthermore, we demonstrate that even supervised fine-tuning PLMs can serve as a comparative standard for addressing downstream domain prediction task.

However, the prediction accuracy achieved in this research is not high, potentially due to the rigorous exclusion of homologous sequences during data preparation. This result suggests that predicting ubiquitination site using only sequence data is challenging, and future directions may involve integrating multimodal data to construct domain-aware model [40,41].

## 5. Conclusion

In summary, we present EUP, a novel application of a large-scale foundation model for predicting ubiquitination, balancing both speed and accuracy. By utilizing cVAE for dimensionality reduction and feature reconstruction, the model significantly improves evaluation accuracy while maintaining efficient inference speed. EUP also incorporates a feature attribution algorithm, which enhances model interpretability by revealing key features’ contributions to ubiquitination prediction across species. By leveraging ESM2’s rich representations of amino acid sequences, EUP captures evolutionary patterns, enhancing its predictive accuracy. Available as a user-friendly web server, EUP provides an accessible tool for predicting ubiquitination sites, offering valuable support to researchers across various domains.

## Supporting information

S1 Fig Multi-species Ubiquitination Site Distribution. (PDF)

S2 Fig Summary Figures for Ten Species. Integrated Gradients (IG) analysis for ubiquitination site predictions in 10 species, encompassing: A summary plot showing the IG values’ impact on the output of the cVAE_ResDNNModel for the top-ranked features. Features are ordered by importance, with red and blue indicating high and low feature values, respectively. K_feature_1542 is identified as the most significant contributor.
(PDF)

S3 Fig Dependence Plots, Ridge Plots, and Swarm Plots for Animal Species. Integrated Gradients (IG) analysis for ubiquitination site predictions in Animal Species, encompassing: (a) Dependence plots for the top four most important features, illustrating the relationship between feature values and IG values. Histograms along the axes summarize the distribution of feature values.(b) A ridge plot displaying the density distribution of the top four features’ values, highlighting variations in feature value distributions.(c) A swarm plot of log-transformed IG values for the top four features, accompanied by Mann-Whitney U tests for statistical significance. The median log-transformed IG values and p-values indicate distinct patterns in feature importance, with K_feature_1542 showing the highest impact.
(PDF)

S4 Fig Dependence Plots, Ridge Plots, and Swarm Plots for Plant Species. Integrated Gradients (IG) analysis for ubiquitination site predictions in Plant Species, encompassing: (a) Dependence plots for the top four most important features, illustrating the relationship between feature values and IG values. Histograms along the axes summarize the distribution of feature values.(b) A ridge plot displaying the density distribution of the top four features’ values, highlighting variations in feature value distributions.(c) A swarm plot of log-transformed IG values for the top four features, accompanied by Mann-Whitney U tests for statistical significance. The median log-transformed IG values and p-values indicate distinct patterns in feature importance, with K_feature_1542 showing the highest impact.
(PDF)

S5 Fig Dependence Plots, Ridge Plots, and Swarm Plots for Microbial Species. Integrated Gradients (IG) analysis for ubiquitination site predictions in Microbial Species, encompassing: (a) Dependence plots for the top four most important features, illustrating the relationship between feature values and IG values. Histograms along the axes summarize the distribution of feature values.(b) A ridge plot displaying the density distribution of the top four features’ values, highlighting variations in feature value distributions.(c) A swarm plot of log-transformed IG values for the top four features, accompanied by Mann-Whitney U tests for statistical significance. The median log-transformed IG values and p-values indicate distinct patterns in feature importance, with K_feature_1542 showing the highest impact.
(PDF)

S6 Fig Comparison of LIME and SHAP interpretability methods for evaluating feature importance in ubiquitination sites across 10 species. The x-axis represents LIME importance scores, while the y-axis represents SHAP importance scores. Blue scatter points show the distribution of feature scores under both methods. The red fitted line (upward slope) and pink confidence interval indicate a significant positive correlation (p < 0.05), though SHAP values are generally higher in absolute scale than LIME (most points lie below the line). While the two methods show high consistency in feature ranking (e.g., the cluster of high-importance features in the upper right), the numerical differences suggest that SHAP is more sensitive to key features.
(PDF)

S7 Fig Spearman correlation heatmap comparing feature importance scores in ubiquitination sites across 10 species between LIME and SHAP interpretability methods. The diagonal represents perfect self-correlation for each method, while the off-diagonal values demonstrate near-perfect rank correlation between LIME and SHAP importance rankings (p < 0.01, statistically significant). The color gradient (blue = 1.00 to red = -1.00) confirms strong positive agreement (uniformly blue tones) with no negative correlations observed. This indicates that while absolute score scales may differ (as seen in scatter plots), both methods consistently identify the same features as most/least important across analyses.
(PDF)

S1 Table Four Model Predictive Evaluation by beta. (PDF)

S2 Table Four Model Predictive Evaluation with DT/FPR. (PDF)

S3 Table Statistical Significance of AUROC Differences Between Models Based on DeLong Test. (PDF)

S4 Table Four Model Predictive Evaluation with tow feature extraction method and Denoising. (PDF)

S5 Table Four Model Predictive Evaluation with tow feature extraction method and Denoising (Independent Validation Set). (PDF)

S6 Table Performance of Models with All Training Strategies on Single Species Data. (PDF)

## References

[1] Zhou J, Xu Y, Lin S, Guo Y, Deng W, Zhang Y, et al. iUUCD 2.0: an update with rich annotations for ubiquitin and ubiquitin-like conjugations. Nucleic Acids Res. 2018;46(D1):D447–53. doi: 10.1093/nar/gkx1041 29106644 PMC5753239

[2] Simoneschi D, Rona G, Zhou N, Jeong Y-T, Jiang S, Milletti G, et al. CRL4AMBRA1 is a master regulator of D-type cyclins. Nature. 2021;592(7856):789–93. doi: 10.1038/s41586-021-03445-y 33854235 PMC8875297

[3] Hofmann R, Akimoto G, Wucherpfennig TG, Zeymer C, Bode JW. Lysine acylation using conjugating enzymes for site-specific modification and ubiquitination of recombinant proteins. Nat Chem. 2020;12(11):1008–15. 32929246 10.1038/s41557-020-0528-y

[4] McManus FP, Lamoliatte F, Thibault P. Identification of cross talk between SUMOylation and ubiquitylation using a sequential peptide immunopurification approach. Nat Protoc. 2017;12(11):2342–58. doi: 10.1038/nprot.2017.105 29048423

[5] Paluda A, Middleton AJ, Rossig C, Mace PD, Day CL. Ubiquitin and a charged loop regulate the ubiquitin E3 ligase activity of Ark2C. Nat Commun. 2022;13(1):1181. doi: 10.1038/s41467-022-28782-y 35246518 PMC8897509

[6] Luo Y, Jiang J, Zhu J, Huang Q, Li W, Wang Y, et al. A Caps-Ubi Model for Protein Ubiquitination Site Prediction. Front Plant Sci. 2022;13:884903. doi: 10.3389/fpls.2022.884903 35693166 PMC9175003

[7] Hitchcock AL, Auld K, Gygi SP, Silver PA. A subset of membrane-associated proteins is ubiquitinated in response to mutations in the endoplasmic reticulum degradation machinery. Proc Natl Acad Sci U S A. 2003;100(22):12735–40. doi: 10.1073/pnas.2135500100 14557538 PMC240687

[8] Wang C, Tan X, Tang D, Gou Y, Han C, Ning W, et al. GPS-Uber: a hybrid-learning framework for prediction of general and E3-specific lysine ubiquitination sites. Brief Bioinform. 2022;23(2):bbab574. doi: 10.1093/bib/bbab574 35037020

[9] Zhang W, Tan X, Lin S, Gou Y, Han C, Zhang C, et al. CPLM 4.0: an updated database with rich annotations for protein lysine modifications. Nucleic Acids Res. 2022;50(D1):D451–9. doi: 10.1093/nar/gkab849 34581824 PMC8728254

[10] Zhang Y-J, Luo Z, Sun Y, Liu J, Chen Z. From beasts to bytes: Revolutionizing zoological research with artificial intelligence. Zool Res. 2023;44(6):1115–31. doi: 10.24272/j.issn.2095-8137.2023.263 37933101 PMC10802096

[11] Wang W, Zhang Y, Liu D, Zhang H, Wang X, Zhou Y. PseAraUbi: predicting arabidopsis ubiquitination sites by incorporating the physico-chemical and structural features. Plant Mol Biol. 2022;110(1–2):81–92. doi: 10.1007/s11103-022-01288-3 35773617

[12] Wang J-R, Huang W-L, Tsai M-J, Hsu K-T, Huang H-L, Ho S-Y. ESA-UbiSite: accurate prediction of human ubiquitination sites by identifying a set of effective negatives. Bioinformatics. 2017;33(5):661–8. doi: 10.1093/bioinformatics/btw701 28062441

[13] Chen Z, Zhou Y, Song J, Zhang Z. hCKSAAP_UbSite: improved prediction of human ubiquitination sites by exploiting amino acid pattern and properties. Biochim Biophys Acta. 2013;1834(8):1461–7. doi: 10.1016/j.bbapap.2013.04.006 23603789

[14] Chen J, Zhao J, Yang S, Chen Z, Zhang Z. Prediction of Protein Ubiquitination Sites in Arabidopsis thaliana. CBIO. 2019;14(7):614–20. doi: 10.2174/1574893614666190311141647

[15] Chen Z, Chen Y-Z, Wang X-F, Wang C, Yan R-X, Zhang Z. Prediction of ubiquitination sites by using the composition of k-spaced amino acid pairs. PLoS One. 2011;6(7):e22930. doi: 10.1371/journal.pone.0022930 21829559 PMC3146527

[16] Lin Z, Akin H, Rao R, Hie B, Zhu Z, Lu W, et al. Evolutionary-scale prediction of atomic-level protein structure with a language model. Science. 2023;379(6637):1123–30. doi: 10.1126/science.ade2574 36927031

[17] Brandes N, Ofer D, Peleg Y, Rappoport N, Linial M. ProteinBERT: a universal deep-learning model of protein sequence and function. Bioinformatics. 2022;38(8):2102–10. doi: 10.1093/bioinformatics/btac020 35020807 PMC9386727

[18] Vaswani A. Attention is all you need. In: Advances in Neural Information Processing Systems, 2017.

[19] Zang J, Chen Y, Liu C, Hu L, Zhao H, Ding W, et al. Genetic code expansion reveals aminoacylated lysine ubiquitination mediated by UBE2W. Nat Struct Mol Biol. 2023;30(1):62–71. doi: 10.1038/s41594-022-00866-9 36593310

[20] He K, Zhang X, Ren S, Sun J. Deep residual learning for image recognition. In: Proceedings of the IEEE Conference on Computer Vision and Pattern Recognition, 2016.

[21] Kingma DP. Auto-encoding variational bayes. arXiv preprint. 2013. doi: 10.48550/arXiv.1312.6114

[22] Luo Z, Wang R, Sun Y, Liu J, Chen Z, Zhang Y-J. Interpretable feature extraction and dimensionality reduction in ESM2 for protein localization prediction. Brief Bioinform. 2024;25(2):bbad534. doi: 10.1093/bib/bbad534 38279650 PMC10818170

[23] Chen H, Lu Y, Dai Z, Yang Y, Li Q, Rao Y. Comprehensive single-cell RNA-seq analysis using deep interpretable generative modeling guided by biological hierarchy knowledge. Brief Bioinform. 2024;25(4):bbae314. doi: 10.1093/bib/bbae314 38960404 PMC11221887

[24] Hayes T, Rao R, Akin H, Sofroniew NJ, Oktay D, Lin Z, et al. Simulating 500 million years of evolution with a language model. Science. 2025;387(6736):850–8. doi: 10.1126/science.ads0018 39818825

[25] Li W, Godzik A. Cd-hit: a fast program for clustering and comparing large sets of protein or nucleotide sequences. Bioinformatics. 2006;22(13):1658–9. doi: 10.1093/bioinformatics/btl158 16731699

[26] Akiba T, Sano S, Yanase T, Ohta T, Koyama M. Optuna: A next-generation hyperparameter optimization framework. In: Proceedings of the 25th ACM SIGKDD international conference on knowledge discovery & data mining, 2019.

[27] Agustianto K, Destarianto P. Imbalance data handling using neighborhood cleaning rule (NCL) sampling method for precision student modeling. In: 2019.

[28] Chicco D, Jurman G. The Matthews correlation coefficient (MCC) should replace the ROC AUC as the standard metric for assessing binary classification. BioData Min. 2023;16(1):4. 36800973 10.1186/s13040-023-00322-4 PMC9938573

[29] Fang Y, Xu F, Wei L, Jiang Y, Chen J, Wei L, et al. AFP-MFL: accurate identification of antifungal peptides using multi-view feature learning. Brief Bioinform. 2023;24(1):bbac606. doi: 10.1093/bib/bbac606 36631407

[30] Sundararajan M, Taly A, Yan Q. Axiomatic Attribution for Deep Networks. In: Proceedings of the 34th International Conference on Machine Learning, 2017. 3319–28.

[31] Luo Z, Wang R, Sun Y, Liu J, Chen Z, Zhang Y-J. Interpretable feature extraction and dimensionality reduction in ESM2 for protein localization prediction. Brief Bioinform. 2024;25(2):bbad534. doi: 10.1093/bib/bbad534 38279650 PMC10818170

[32] Sun X, Xu W. Fast Implementation of DeLong’s Algorithm for Comparing the Areas Under Correlated Receiver Operating Characteristic Curves. IEEE Signal Process Lett. 2014;21(11):1389–93. doi: 10.1109/lsp.2014.2337313

[33] Guidotti R, Monreale A, Ruggieri S, Turini F, Giannotti F, Pedreschi D. A Survey of Methods for Explaining Black Box Models. ACM Comput Surv. 2018;51(5):1–42. doi: 10.1145/3236009

[34] Mishra S, Sturm BL, Dixon S. Local interpretable model-agnostic explanations for music content analysis. In: 2017.

[35] Zhang J, Wysocki R, Li F, Yu M, Martinoia E, Song W-Y. Role of ubiquitination in arsenic tolerance in plants. Trends Plant Sci. 2023;28(8):880–92. doi: 10.1016/j.tplants.2023.03.008 37002000

[36] Lindsey J, Gurnee W, Ameisen E, Chen B, Pearce A, Turner NL. On the biology of a large language model. Transformer Circuits Thread. 2025.

[37] Detlefsen NS, Hauberg S, Boomsma W. Learning meaningful representations of protein sequences. Nat Commun. 2022;13(1):1914. doi: 10.1038/s41467-022-29443-w 35395843 PMC8993921

[38] Hayes T, Rao R, Akin H, Sofroniew NJ, Oktay D, Lin Z, et al. Simulating 500 million years of evolution with a language model. bioRxiv. 2024. doi: 10.1101/2024.07.01.600583 39818825

[39] Avraham O, Tsaban T, Ben-Aharon Z, Tsaban L, Schueler-Furman O. Protein language models can capture protein quaternary state. BMC Bioinformatics. 2023;24(1):433. doi: 10.1186/s12859-023-05549-w 37964216 PMC10647083

[40] Smith J, Shi Y, Benedikt M, Nikolic M. Scalable analysis of multi-modal biomedical data. Gigascience. 2021;10(9):giab058. doi: 10.1093/gigascience/giab058 34508579 PMC8434767

[41] Fang Y, Jiang Y, Wei L, Ma Q, Ren Z, Yuan Q, et al. DeepProSite: structure-aware protein binding site prediction using ESMFold and pretrained language model. Bioinformatics. 2023;39(12):btad718. doi: 10.1093/bioinformatics/btad718 38015872 PMC10723037

