# Supplementary figures and images for: EUP: Enhanced cross-species prediction of ubiquitination sites via a conditional variational autoencoder network based on ESM2
Source: PLoS Comput Biol. 2025 Jul 16;21(7):e1013268. doi: 10.1371/journal.pcbi.1013268 (PMC12266453; doi:10.1371/journal.pcbi.1013268)

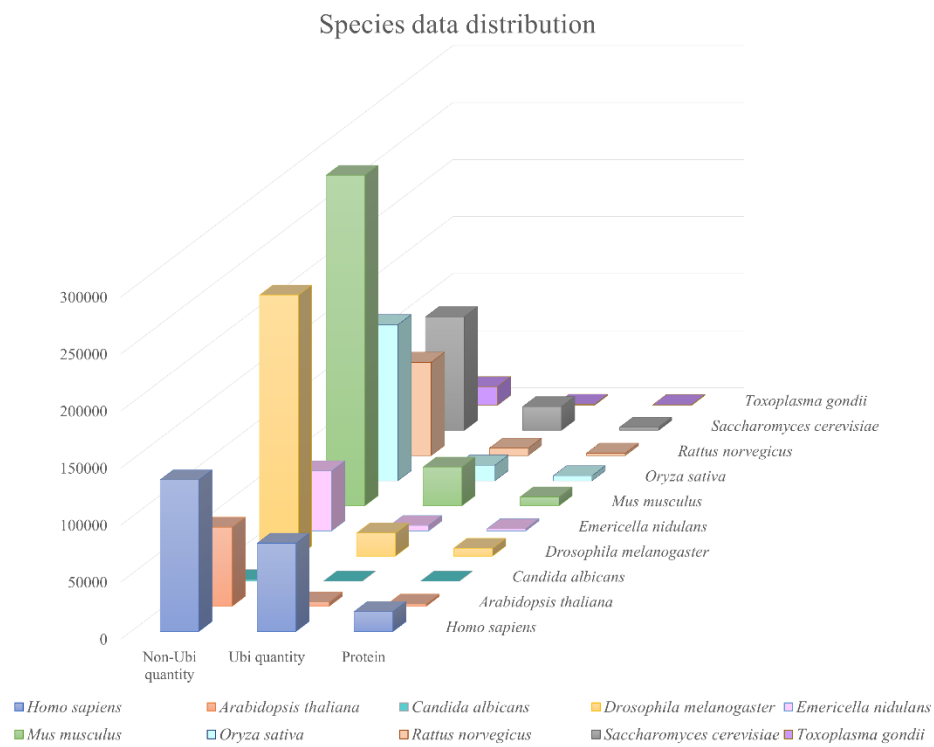

**S1 Fig. Multi-species Ubiquitination Site Distribution**

Supplement: S1 Fig — (PDF) [file pcbi.1013268.s001.pdf]
