# Supplementary material for: EUP: Enhanced cross-species prediction of ubiquitination sites via a conditional variational autoencoder network based on ESM2
Source: PLoS Comput Biol. 2025 Jul 16;21(7):e1013268. doi: 10.1371/journal.pcbi.1013268 (PMC12266453; doi:10.1371/journal.pcbi.1013268)
Supplement: S2 Fig — Integrated Gradients (IG) analysis for ubiquitination site predictions in 10 species, encompassing: A summary plot showing the IG values’ impact on the output of the cVAE_ResDNNModel for the top-ranked features. Features are ordered by importance, with red and blue indicating high and low feature values, respectively. K_feature_1542 is identified as the most significant contributor. (PDF) [file pcbi.1013268.s002.pdf]

1. *Arabidopsis thaliana*

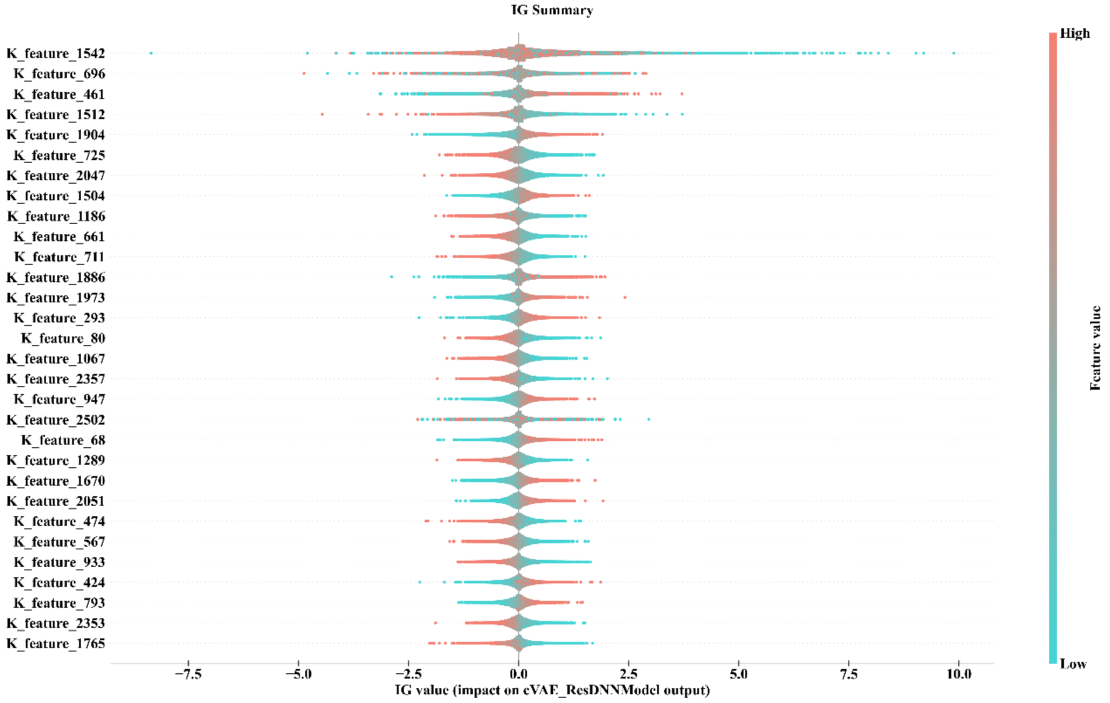

2. *Candida albicans*

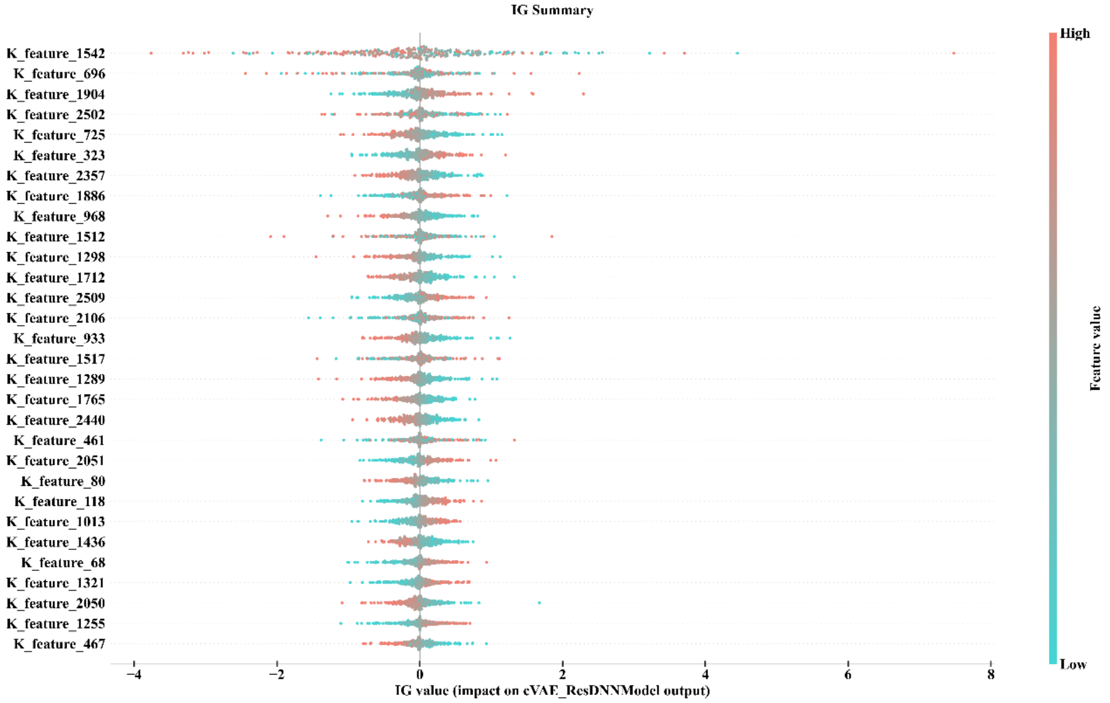

3. Drosophila melanogaster

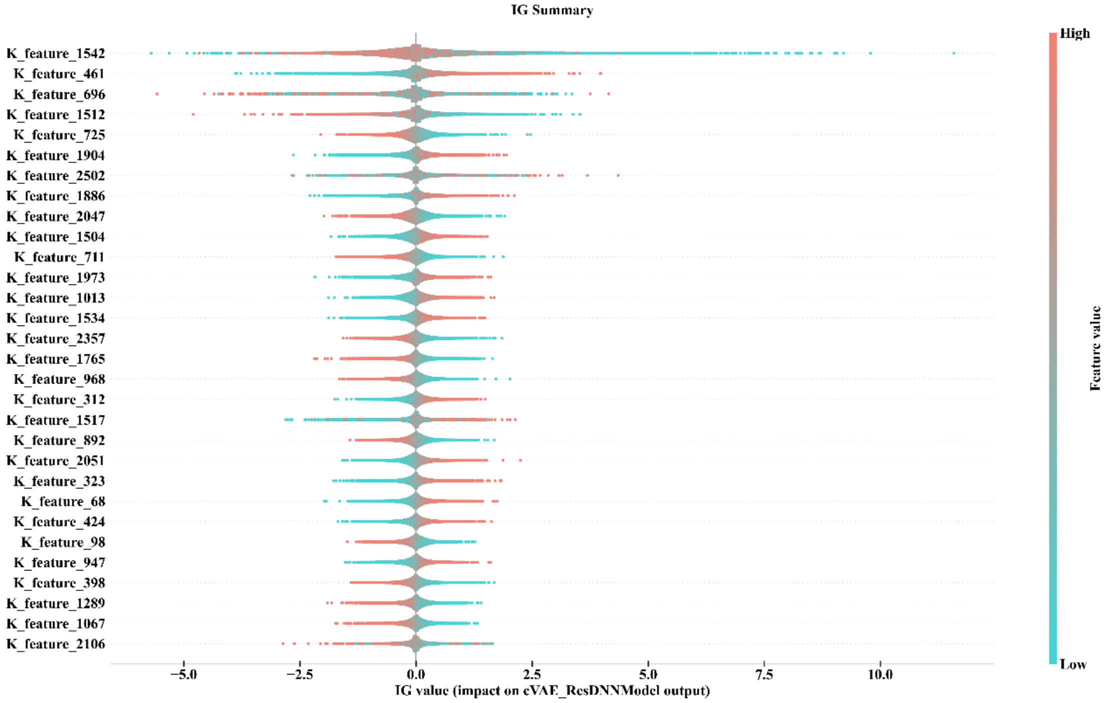

4. Emericella nidulans

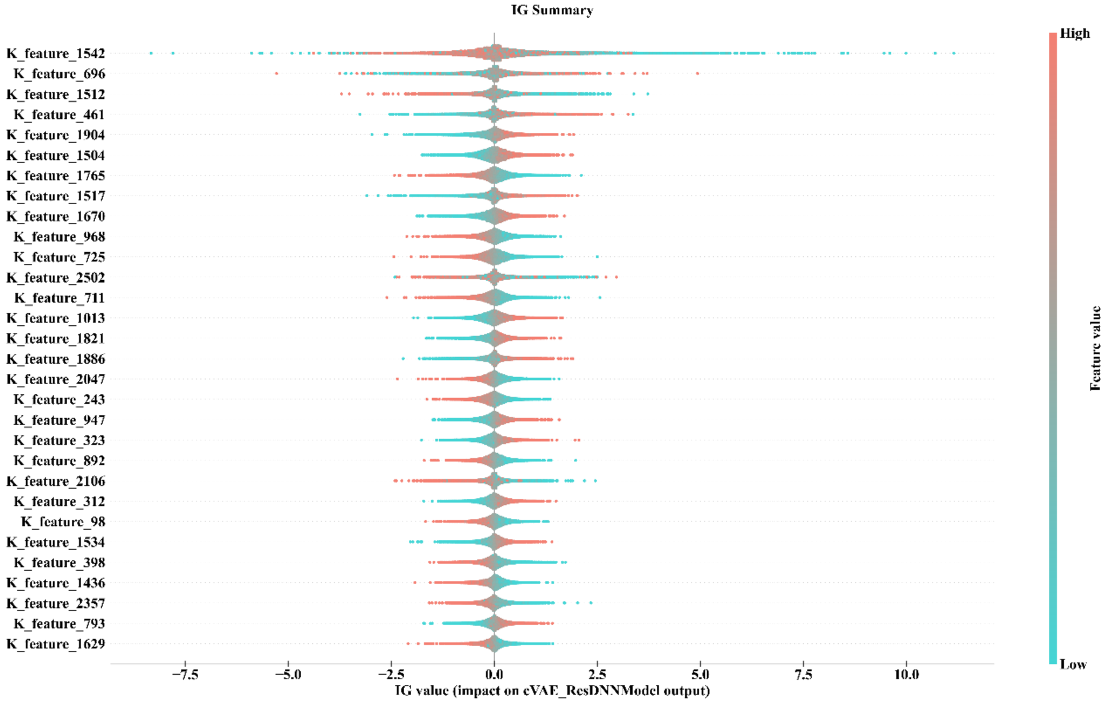

5. *Homo sapiens*

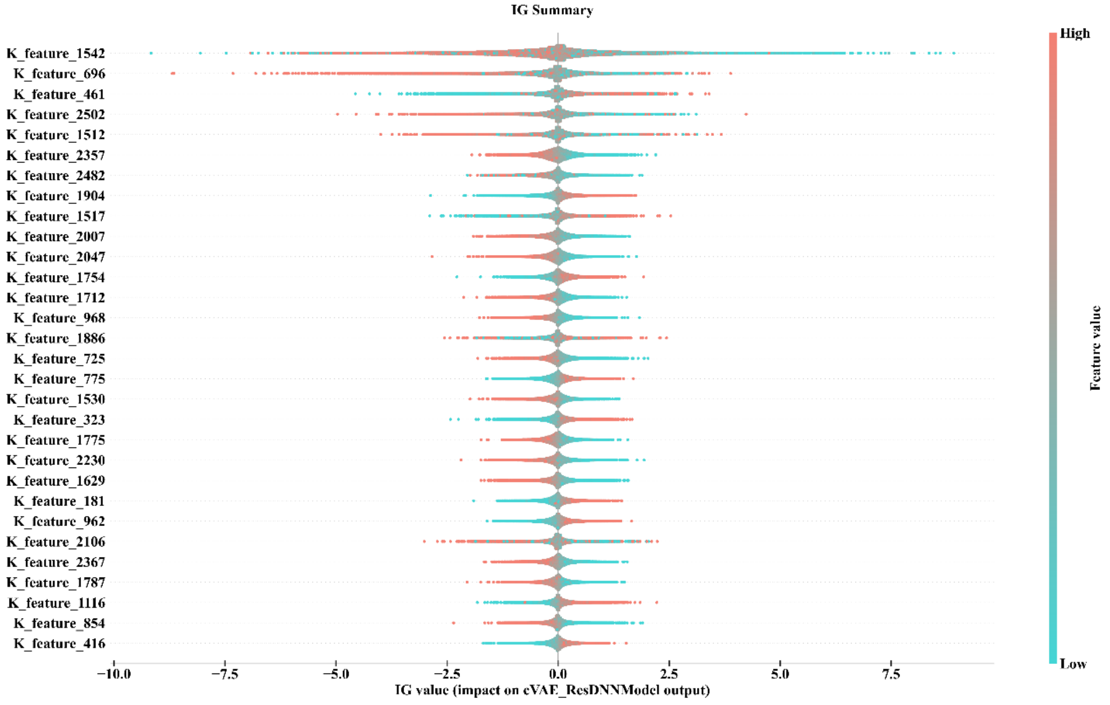

6. *Mus musculus*

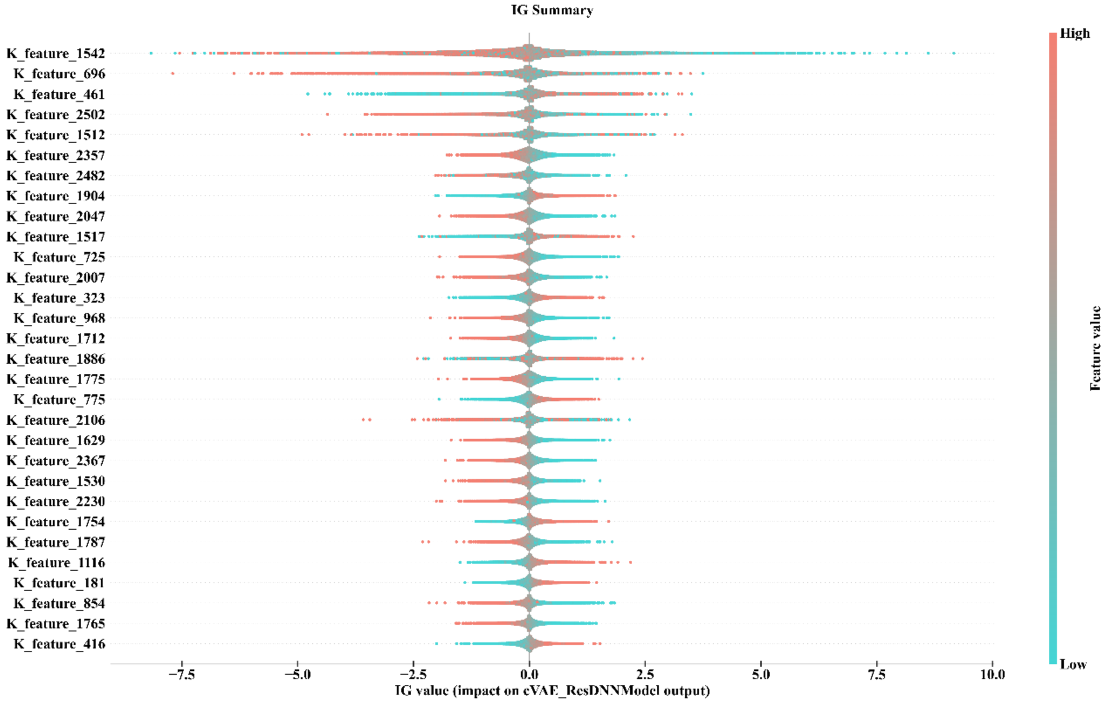

7. Oryza sativa

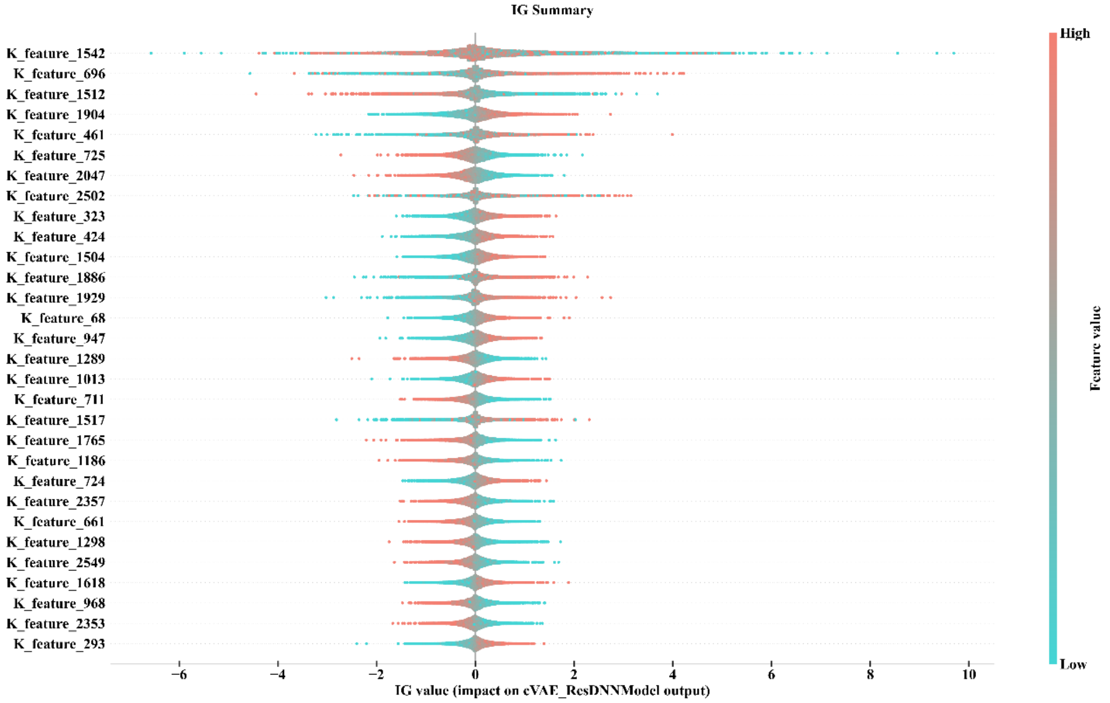

8. Rattus norvegicus

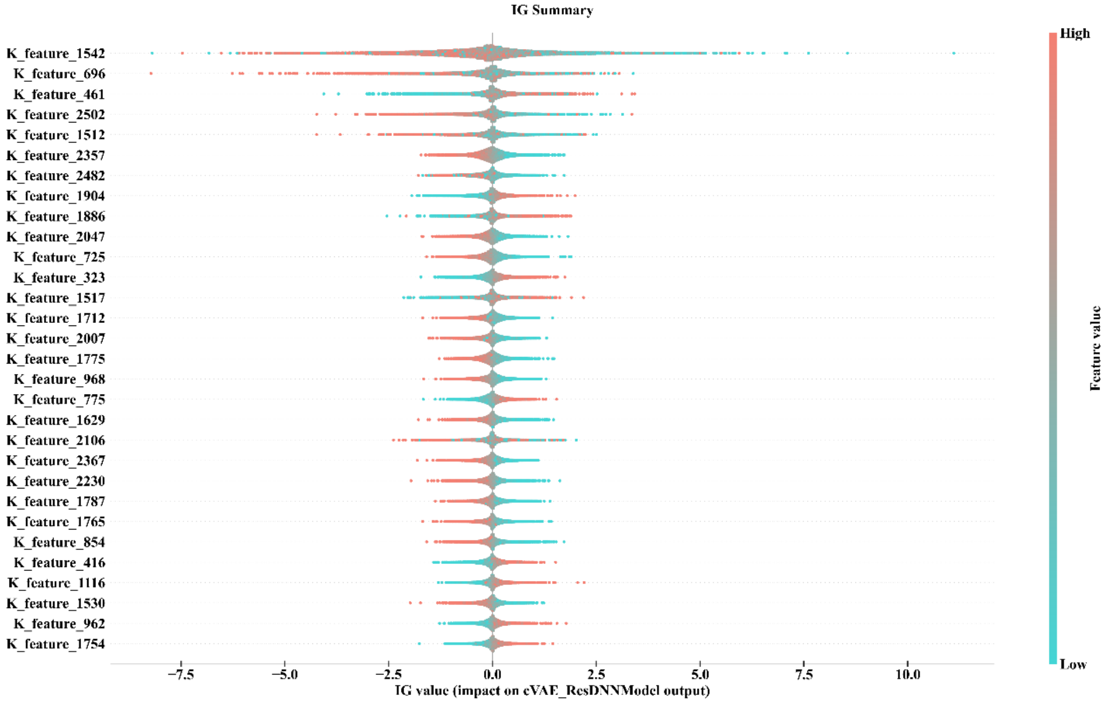

9. *Saccharomyces cerevisiae*

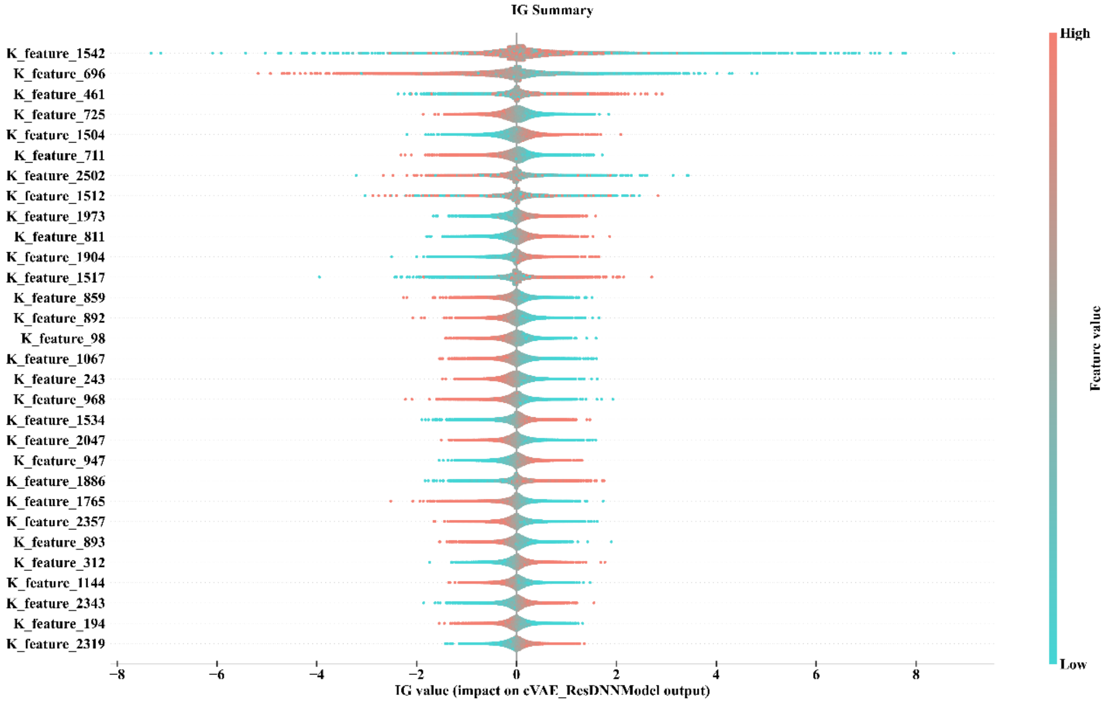

10. *Toxoplasma_gondii*

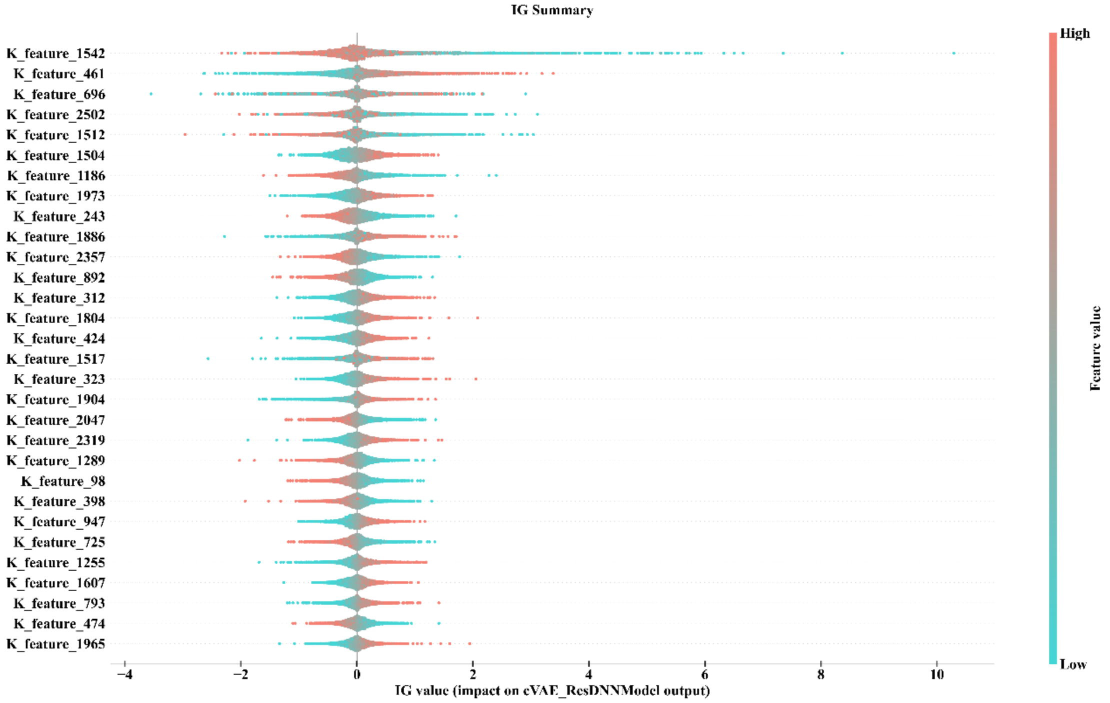

S2 Fig. Summary Figures for Ten Species. Integrated Gradients (IG) analysis for ubiquitination site predictions in 10 species, encompassing: A summary plot showing the IG values' impact on the output of the cVAE_ResDNNModel for the top-ranked features. Features are ordered by importance, with red and blue indicating high and low feature values, respectively. K_feature_1542 is identified as the most significant contributor.
