# Supplementary material for: EUP: Enhanced cross-species prediction of ubiquitination sites via a conditional variational autoencoder network based on ESM2
Source: PLoS Comput Biol. 2025 Jul 16;21(7):e1013268. doi: 10.1371/journal.pcbi.1013268 (PMC12266453; doi:10.1371/journal.pcbi.1013268)
Supplement: S4 Fig — Integrated Gradients (IG) analysis for ubiquitination site predictions in Plant Species, encompassing: (a) Dependence plots for the top four most important features, illustrating the relationship between feature values and IG values. Histograms along the axes summarize the distribution of feature values.(b) A ridge plot displaying the density distribution of the top four features’ values, highlighting variations in feature value distributions.(c) A swarm plot of log-transformed IG values for the top four features, accompanied by Mann-Whitney U tests for statistical significance. The median log-transformed IG values and p-values indicate distinct patterns in feature importance, with K_feature_1542 showing the highest impact. (PDF) [file pcbi.1013268.s004.pdf]

1. Arabidopsis thaliana

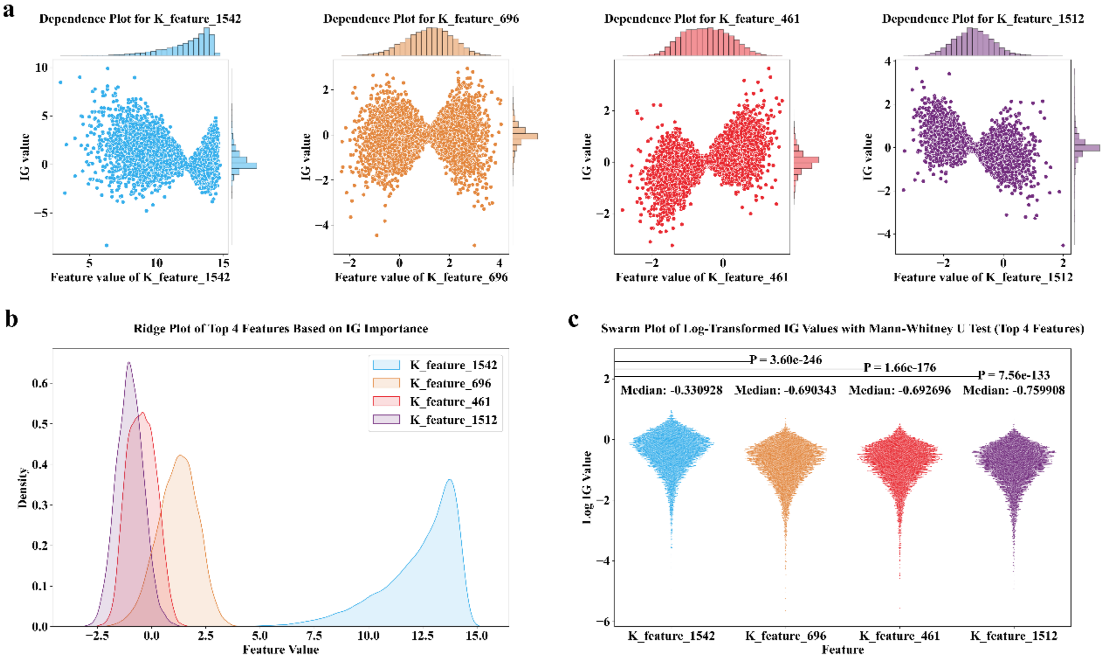

2. Oryza sativa

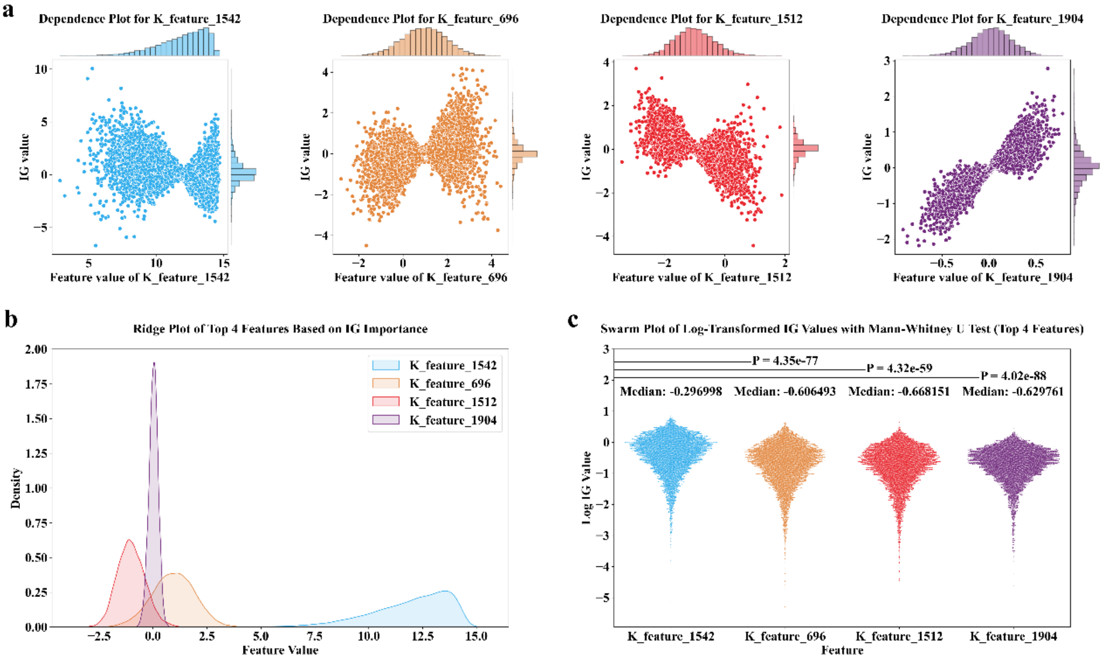

S4 Fig. Dependence Plots, Ridge Plots, and Swarm Plots for Plant Species. Integrated Gradients (IG) analysis for ubiquitination site predictions in Plant Species, encompassing: (a) Dependence plots for the top four most important features, illustrating the relationship between feature values and IG values. Histograms along the axes summarize the distribution of feature values.(b) A ridge plot displaying the density distribution of the top four features' values, highlighting variations in feature value distributions.(c) A swarm plot of log-transformed IG values for the top four features, accompanied by Mann-Whitney U tests for statistical significance. The median log-transformed IG

values and p-values indicate distinct patterns in feature importance, with K_feature_1542 showing the highest impact.
