# Supplementary material for: EUP: Enhanced cross-species prediction of ubiquitination sites via a conditional variational autoencoder network based on ESM2
Source: PLoS Comput Biol. 2025 Jul 16;21(7):e1013268. doi: 10.1371/journal.pcbi.1013268 (PMC12266453; doi:10.1371/journal.pcbi.1013268)
Supplement: S6 Fig — The x-axis represents LIME importance scores, while the y-axis represents SHAP importance scores. Blue scatter points show the distribution of feature scores under both methods. The red fitted line (upward slope) and pink confidence interval indicate a significant positive correlation (p < 0.05), though SHAP values are generally higher in absolute scale than LIME (most points lie below the line). While the two methods show high consistency in feature ranking (e.g., the cluster of high-importance features in the upper right), the numerical differences suggest that SHAP is more sensitive to key features. (PDF) [file pcbi.1013268.s006.pdf]

1. *Arabidopsis thaliana*

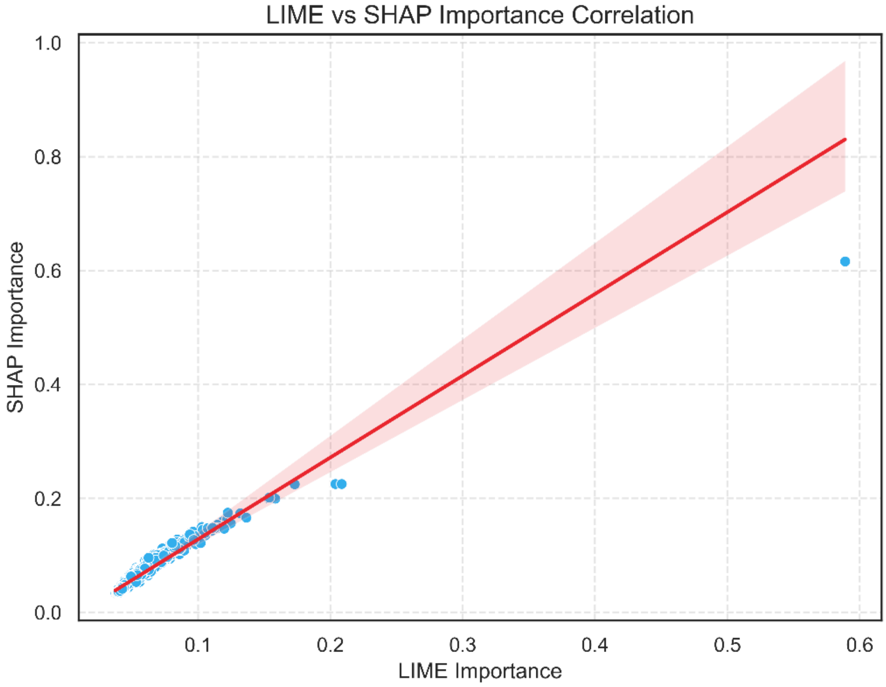

2. *Candida albicans*

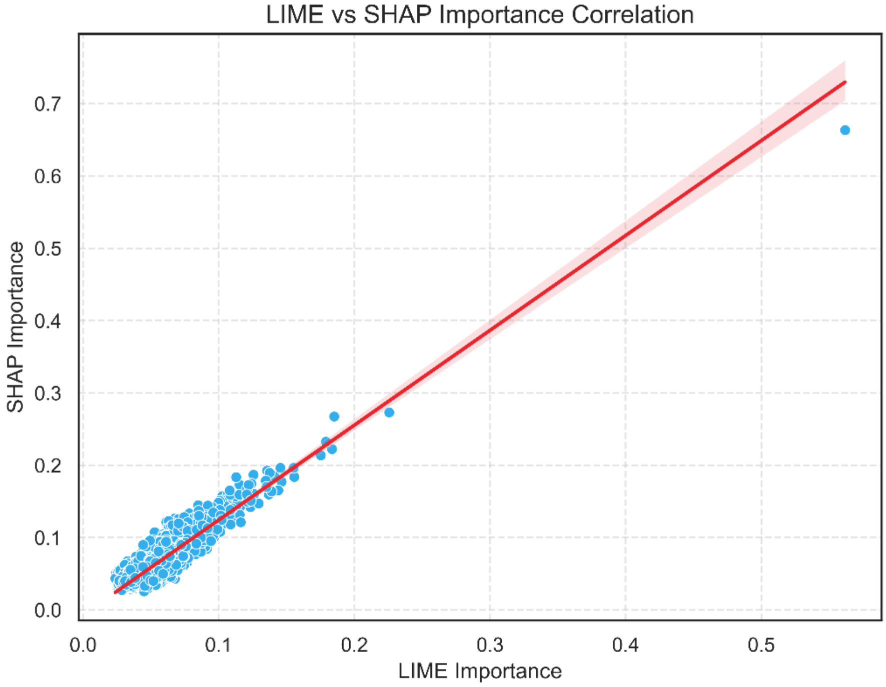

3. *Drosophila melanogaster*

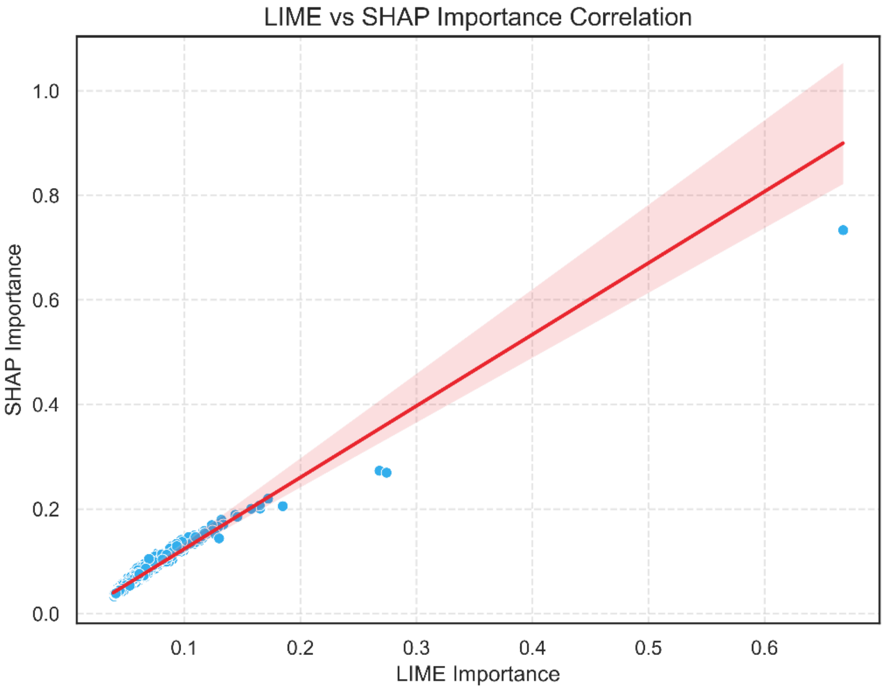

4. *Emericella nidulans*

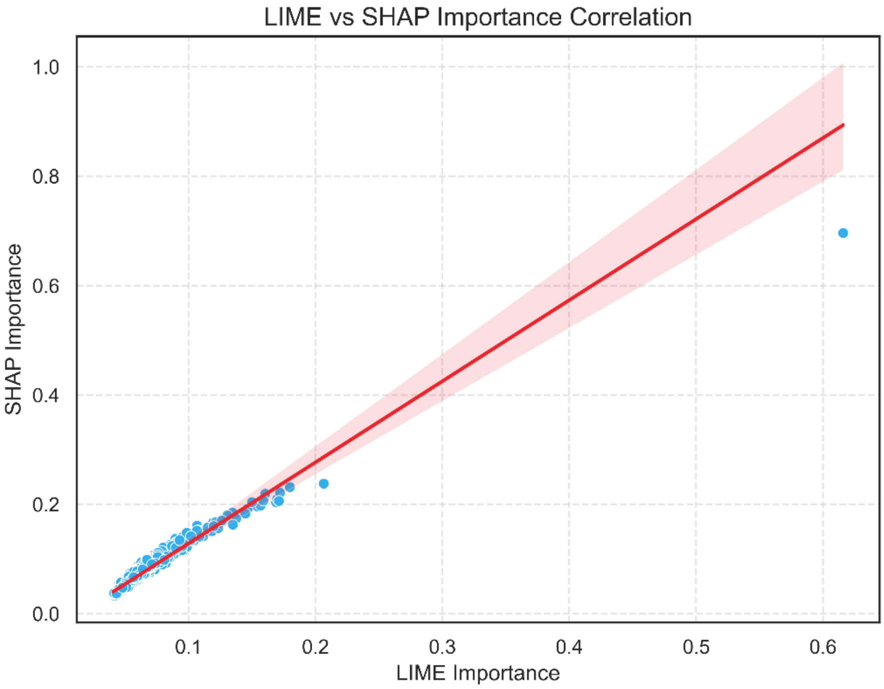

5. *Homo sapiens*

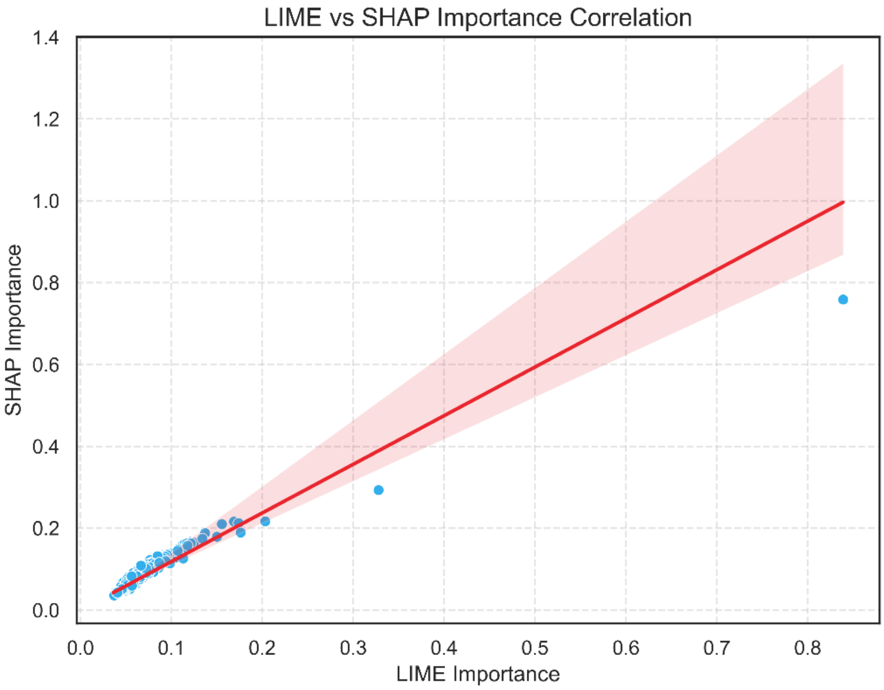

6. *Mus musculus*

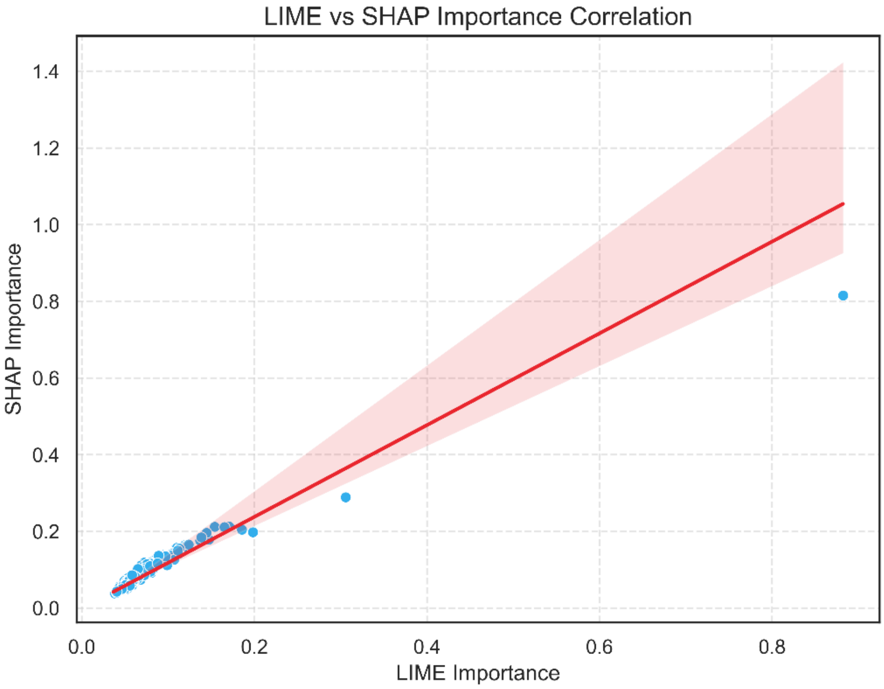

7. *Oryza sativa*

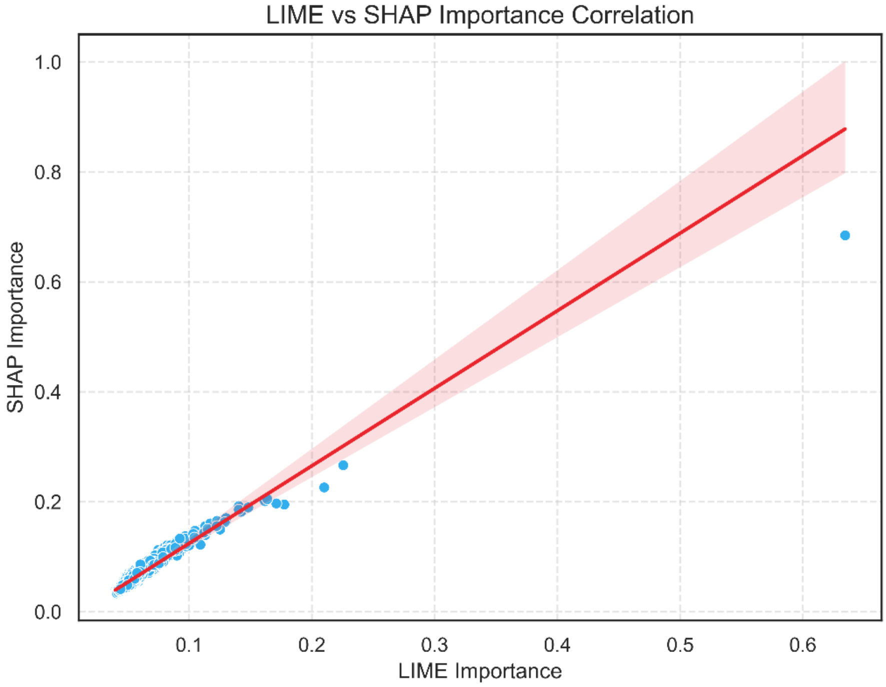

8. *Rattus norvegicus*

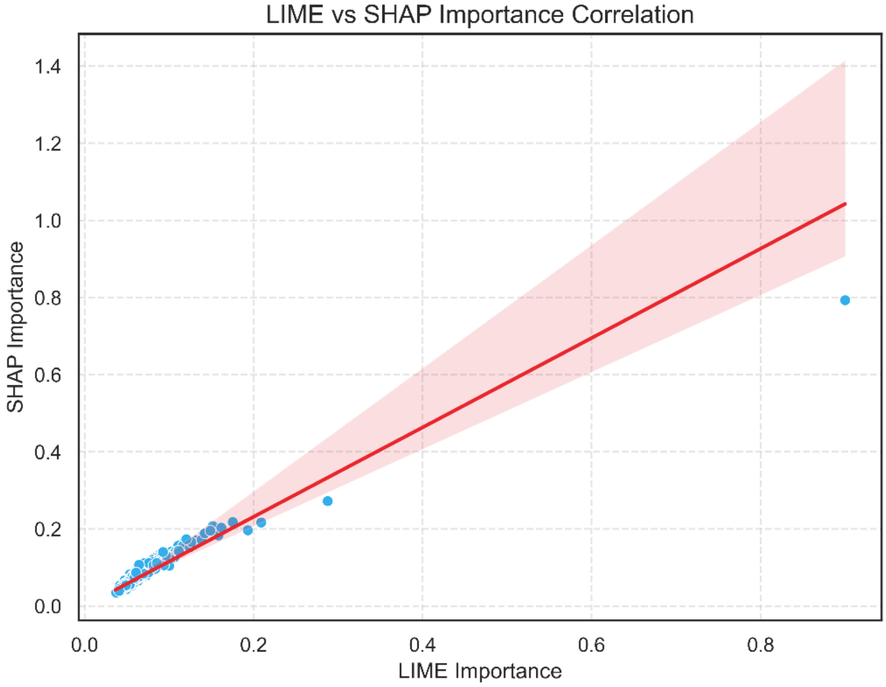

9. *Saccharomyces cerevisiae*

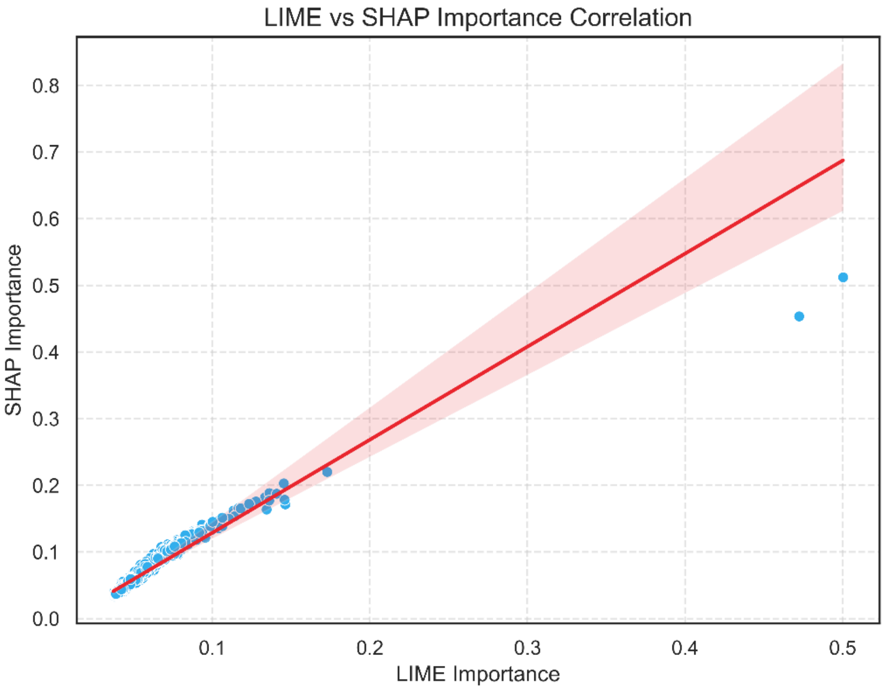

10. *Toxoplasma_gondii*

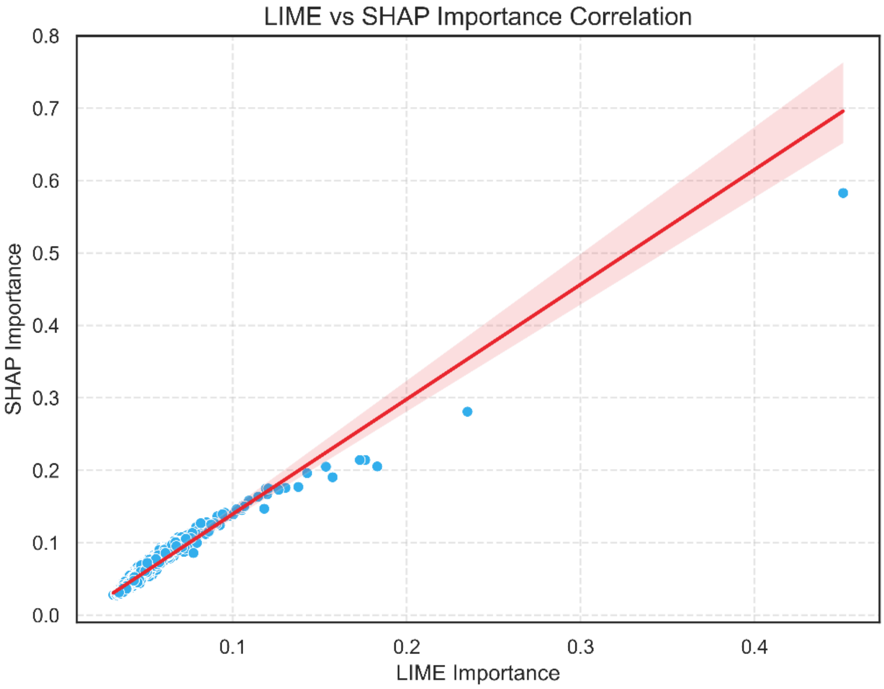

S6 Fig. Comparison of LIME and SHAP interpretability methods for evaluating feature importance in ubiquitination sites across 10 species. The x-axis represents LIME importance scores, while the y-axis represents SHAP importance scores. Blue scatter points show the distribution of feature scores under both methods. The red fitted line (upward slope) and pink confidence interval indicate a significant positive correlation ($p < 0.05$), though SHAP values are generally higher in absolute scale than LIME (most points lie below the line). While the

two methods show high consistency in feature ranking (e.g., the cluster of high-importance features in the upper right), the numerical differences suggest that SHAP is more sensitive to key features.
