# Supplementary material for: EUP: Enhanced cross-species prediction of ubiquitination sites via a conditional variational autoencoder network based on ESM2
Source: PLoS Comput Biol. 2025 Jul 16;21(7):e1013268. doi: 10.1371/journal.pcbi.1013268 (PMC12266453; doi:10.1371/journal.pcbi.1013268)
Supplement: S7 Fig — The diagonal represents perfect self-correlation for each method, while the off-diagonal values demonstrate near-perfect rank correlation between LIME and SHAP importance rankings (p < 0.01, statistically significant). The color gradient (blue = 1.00 to red = -1.00) confirms strong positive agreement (uniformly blue tones) with no negative correlations observed. This indicates that while absolute score scales may differ (as seen in scatter plots), both methods consistently identify the same features as most/least important across analyses. (PDF) [file pcbi.1013268.s007.pdf]

1. *Arabidopsis thaliana*

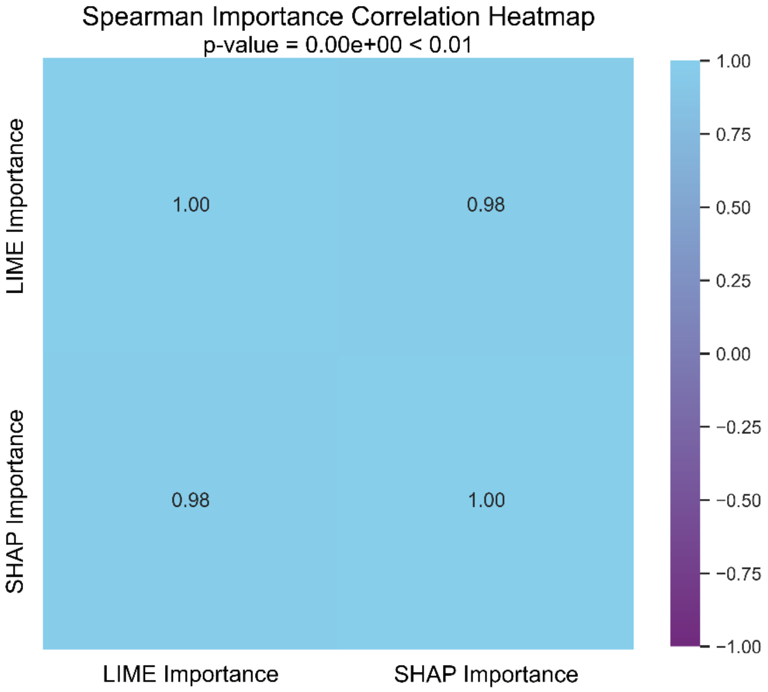

2. *Candida albicans*

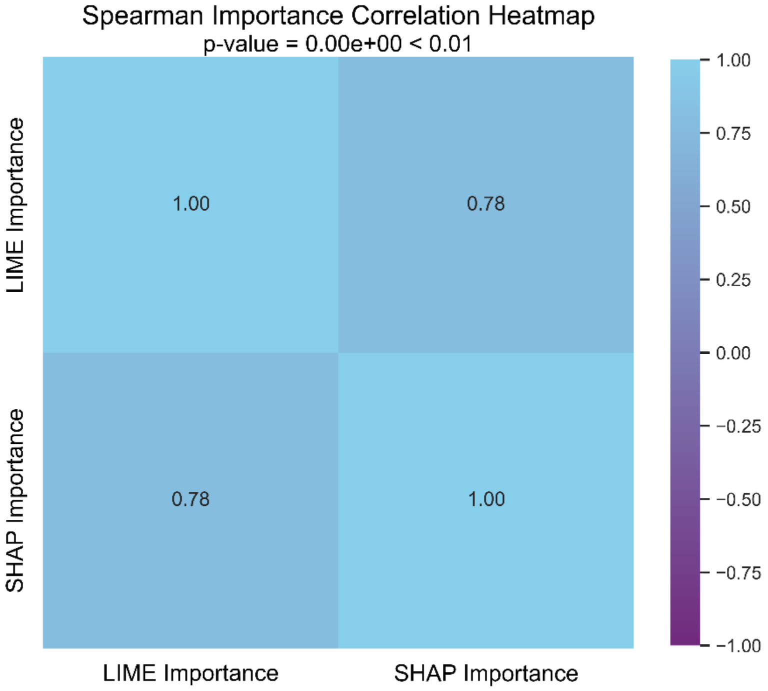

3. *Drosophila melanogaster*

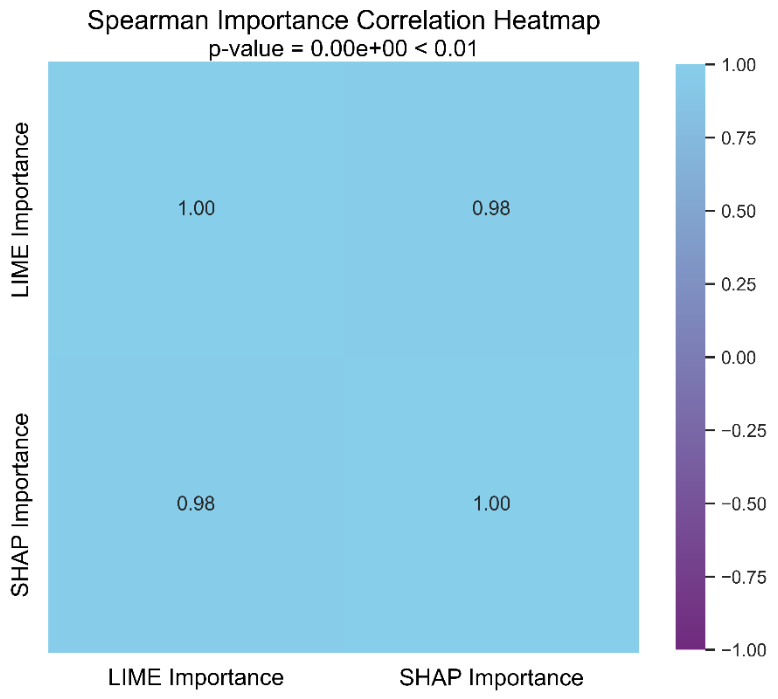

4. *Emericella nidulans*

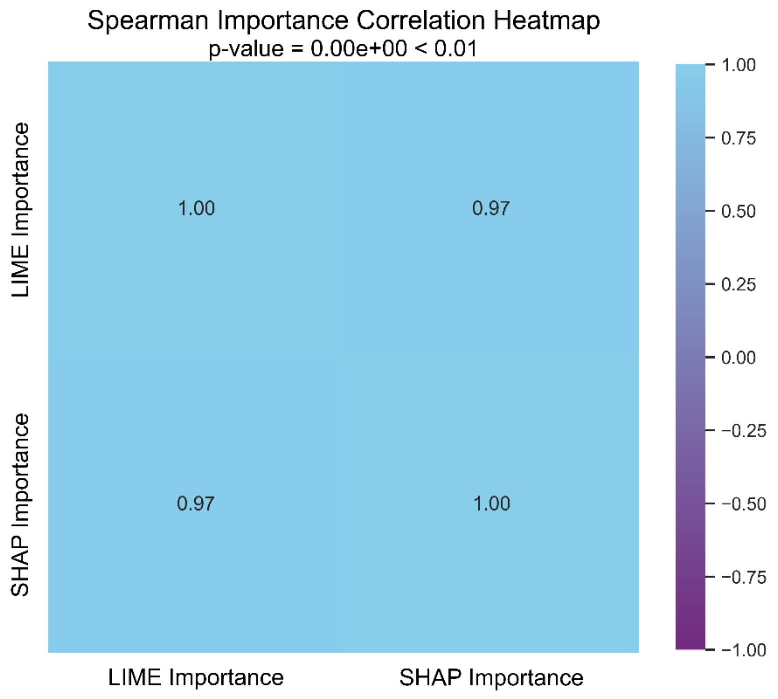

5. *Homo sapiens*

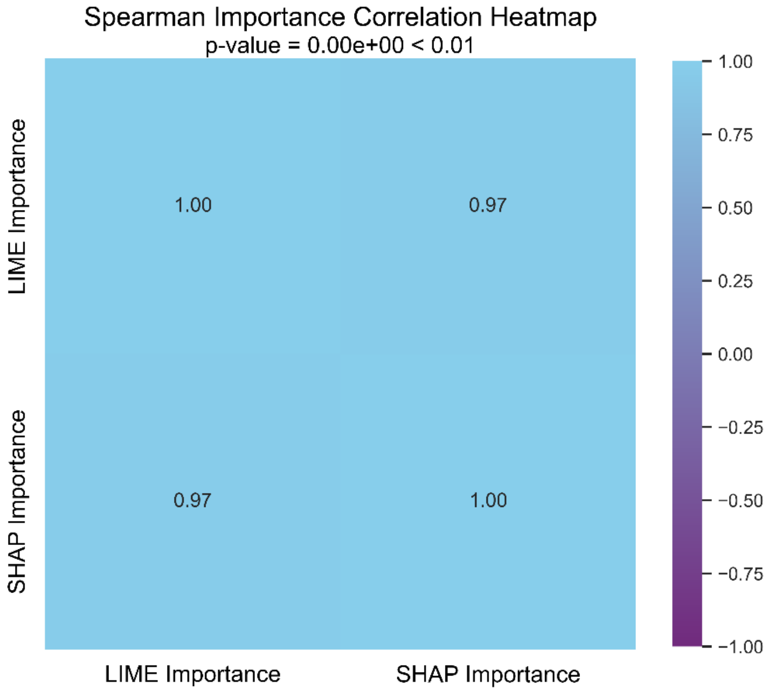

6. *Mus musculus*

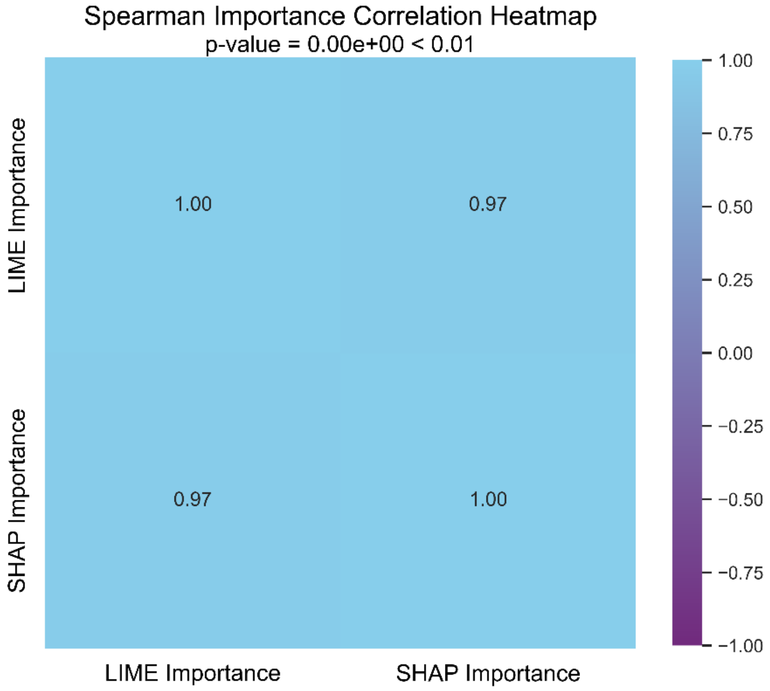

7. *Oryza sativa*

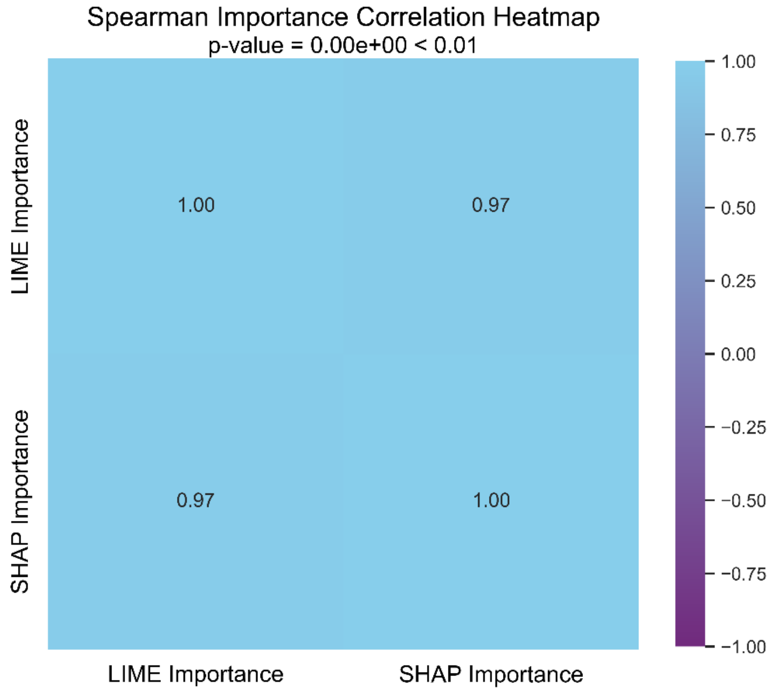

8. *Rattus norvegicus*

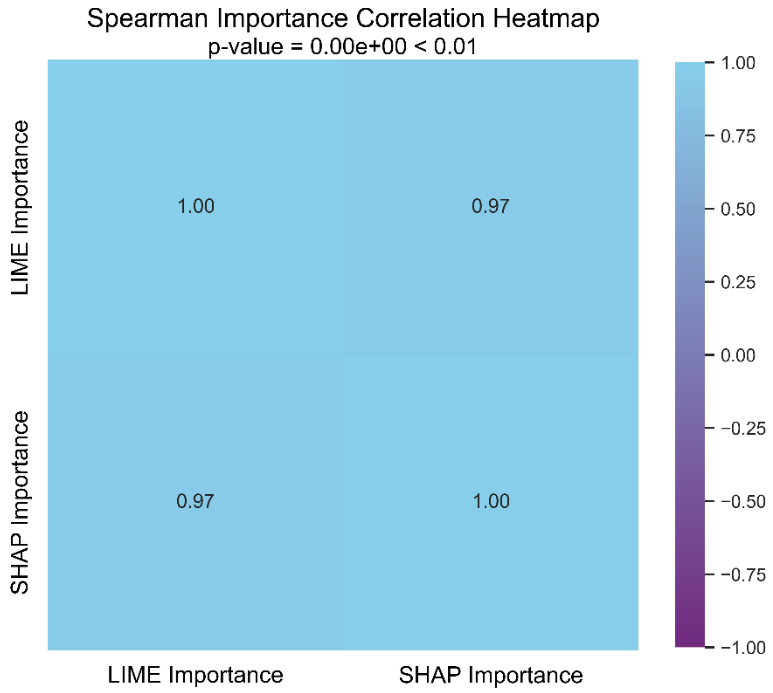

9. *Saccharomyces cerevisiae*

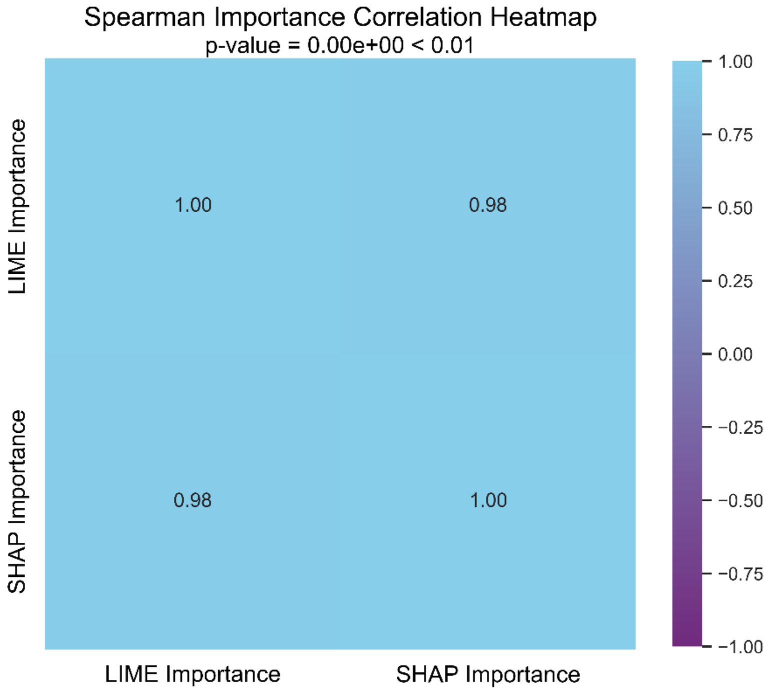

10. *Toxoplasma_gondii*

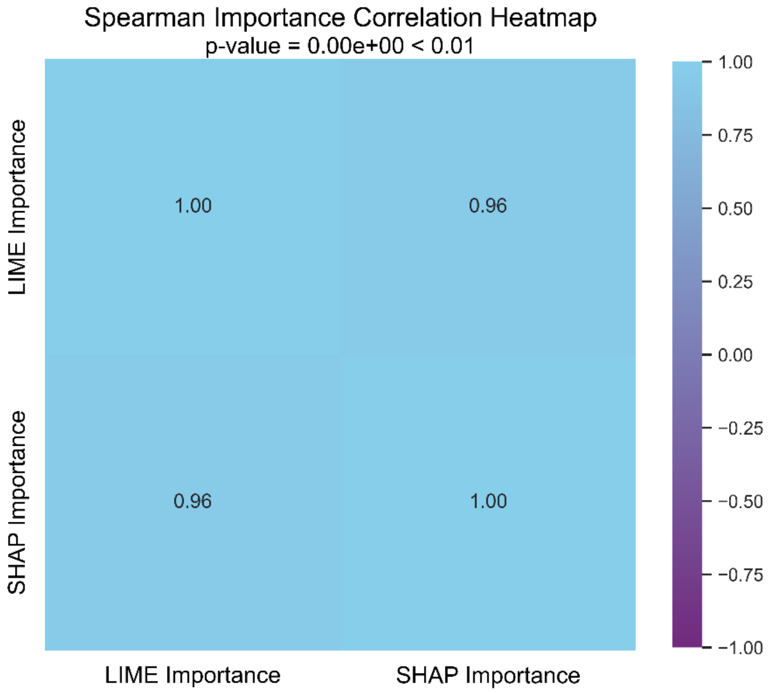

S7 Fig. Spearman correlation heatmap comparing feature importance scores in ubiquitination sites across 10 species between LIME and SHAP interpretability methods. The diagonal represents

perfect self-correlation for each method, while the off-diagonal values demonstrate near-perfect rank correlation between LIME and SHAP importance rankings ($p < 0.01$, statistically significant). The color gradient (blue = 1.00 to red = -1.00) confirms strong positive agreement (uniformly blue tones) with no negative correlations observed. This indicates that while absolute score scales may differ (as seen in scatter plots), both methods consistently identify the same features as most/least important across analyses.
