# Supplementary material for: EUP: Enhanced cross-species prediction of ubiquitination sites via a conditional variational autoencoder network based on ESM2
Source: PLoS Comput Biol. 2025 Jul 16;21(7):e1013268. doi: 10.1371/journal.pcbi.1013268 (PMC12266453; doi:10.1371/journal.pcbi.1013268)
Supplement: S1 Table — (PDF) [file pcbi.1013268.s008.pdf]

S1 Table. Four Model Predictive Evaluation by beta

Model Name	Beta	MCC	F1_Score	Recall	Accuracy	AUC	PR
cVAEResDNN	1	0.255	0.390	0.643	0.686	0.722	0.311
cVAEResDNN	0.3	0.253	0.387	0.488	0.758	0.717	0.309
cVAEDNNLiner	1	0.254	0.389	0.633	0.691	0.708	0.298
cVAEDNNLiner	0.3	0.251	0.386	0.498	0.753	0.710	0.313

In the cVAEResDNN and cVAEDNNLiner models, modifying β to 0.3 primarily affects performance in terms of significant changes in Recall and Accuracy, while other metrics (such as MCC, F1_Score, AUC, and PR) exhibit relatively minor variations. Specifically, setting β=0.3 makes the model more inclined to improve overall accuracy but reduces its ability to identify positive-class samples.

This phenomenon may stem from the following reasons: reducing the β value decreases the weight of the KL divergence, thereby weakening the regularization constraints on the latent space. In this case, the model focuses more on the reconstruction loss ($\mathcal{L}_{REC}$) while inadequately optimizing the distribution of the latent space. This adjustment causes the model to behave more "conservatively" in classification tasks—since negative-class samples dominate the dataset, the model tends to reduce predictions for positive-class samples to improve overall prediction accuracy. As a result, Accuracy increases due to higher prediction accuracy for negative-class samples, while Recall declines due to reduced identification capability for positive-class samples. Other composite metrics (such as MCC and F1_Score), being influenced by both precision and recall, show relatively smaller changes in magnitude.
