# Supplementary material for: EUP: Enhanced cross-species prediction of ubiquitination sites via a conditional variational autoencoder network based on ESM2
Source: PLoS Comput Biol. 2025 Jul 16;21(7):e1013268. doi: 10.1371/journal.pcbi.1013268 (PMC12266453; doi:10.1371/journal.pcbi.1013268)
Supplement: S2 Table — (PDF) [file pcbi.1013268.s009.pdf]

S2 Table. Four Model Predictive Evaluation with DT/FPR

Model Name	DT/FPR	MCC	F1_Score	Recall	Accuracy	AUC	PR
ResDNN	DT=0.4	0.251	0.389	0.562	0.724	0.727	0.325
	FPR=5%	0.198	0.261	0.191	0.832	0.727	0.325
DNNLiner	DT=0.4	0.231	0.374	0.595	0.689	0.712	0.306
	FPR=5%	0.179	0.242	0.175	0.829	0.712	0.306
cVAEResDNN	DT=0.4	0.255	0.390	0.643	0.686	0.722	0.311
	FPR=5%	0.175	0.239	0.172	0.829	0.722	0.316
cVAEDNNLiner	DT=0.4	0.254	0.389	0.633	0.691	0.708	0.298
	FPR=5%	0.154	0.218	0.155	0.826	0.708	0.296
