# Supplementary material for: EUP: Enhanced cross-species prediction of ubiquitination sites via a conditional variational autoencoder network based on ESM2
Source: PLoS Comput Biol. 2025 Jul 16;21(7):e1013268. doi: 10.1371/journal.pcbi.1013268 (PMC12266453; doi:10.1371/journal.pcbi.1013268)
Supplement: S3 Table — (PDF) [file pcbi.1013268.s010.pdf]

S3 Table. Statistical Significance of AUROC Differences Between Models Based on DeLong Test

Model 1	Model 2	Model 1 ROC_AUC	Model 2 ROC_AUC	AUC Difference	p-value	Significant ($p < 0.05$)
DNNLinear	ResDNN	0.712	0.727	0.015	1.23E-50	Yes
DNNLinear	CVAE_DNNLinear	0.712	0.708	0.004	0.006032	Yes
DNNLinear	CVAE_ResDNN	0.712	0.722	0.011	7.51E-14	Yes
ResDNN	CVAE_DNNLinearl	0.727	0.708	0.019	8.50E-46	Yes
ResDNN	CVAE_ResDNN	0.727	0.722	0.005	0.000199	Yes
CVAE_DNNLinear	CVAE_ResDNN	0.708	0.722	0.015	9.91E-35	Yes
