# Supplementary material for: EUP: Enhanced cross-species prediction of ubiquitination sites via a conditional variational autoencoder network based on ESM2
Source: PLoS Comput Biol. 2025 Jul 16;21(7):e1013268. doi: 10.1371/journal.pcbi.1013268 (PMC12266453; doi:10.1371/journal.pcbi.1013268)
Supplement: S4 Table — (PDF) [file pcbi.1013268.s011.pdf]

S4 Table. Four Model Predictive Evaluation with tow feature extraction method and Denoising

Model Name	Feat_extract	DN	MCC	F1_Score	Recall	Accuracy	AUC	PR
ResDNN	ESM2	NCR	0.251	0.389	0.562	0.724	0.727	0.325
		None	0.256	0.391	0.639	0.689	0.732	0.326
	ESMc	NCR	0.244	0.382	0.645	0.674	0.724	0.316
		None	0.242	0.381	0.639	0.676	0.725	0.320
DNNLiner	ESM2	NCR	0.231	0.374	0.595	0.689	0.712	0.306
		None	0.231	0.374	0.600	0.686	0.712	0.306
	ESMc	NCR	0.210	0.358	0.661	0.631	0.696	0.283
		None	0.214	0.362	0.612	0.664	0.699	0.286
cVAEResDNN	ESM2	NCRENN	0.248	0.380	0.449	0.771	0.711	0.306
		NCR	0.255	0.390	0.643	0.686	0.722	0.311
		None	0.251	0.366	0.359	0.806	0.725	0.332
	ESMc	NCRENN	0.231	0.370	0.476	0.748	0.715	0.307
		NCR	0.246	0.383	0.502	0.747	0.725	0.321
		None	0.241	0.365	0.384	0.792	0.722	0.320
cVAEDNNLiner	ESM2	NCRENN	0.246	0.382	0.482	0.756	0.702	0.308
		NCR	0.254	0.389	0.633	0.691	0.708	0.298
		None	0.245	0.369	0.394	0.790	0.714	0.321
	ESMc	NCRENN	0.232	0.373	0.502	0.737	0.707	0.306
		NCR	0.248	0.384	0.494	0.753	0.723	0.319
		None	0.240	0.365	0.387	0.790	0.720	0.321
