# Supplementary material for: EUP: Enhanced cross-species prediction of ubiquitination sites via a conditional variational autoencoder network based on ESM2
Source: PLoS Comput Biol. 2025 Jul 16;21(7):e1013268. doi: 10.1371/journal.pcbi.1013268 (PMC12266453; doi:10.1371/journal.pcbi.1013268)
Supplement: S5 Table — (PDF) [file pcbi.1013268.s012.pdf]

S5 Table. Four Model Predictive Evaluation with tow feature extraction method and Denoising (Independent Validation Set)

Model Name	Feat_extract	DN	MCC	F1_Score	Recall	Accuracy	AUC	PR
ResDNN	ESM2	NCR	0.192	0.628	0.683	0.594	0.603	0.566
		None	0.190	0.639	0.721	0.592	0.623	0.602
	ESMc	NCR	0.217	0.656	0.755	0.604	0.613	0.560
		None	0.219	0.657	0.758	0.605	0.616	0.548
DNNLiner	ESM2	NCR	0.148	0.619	0.695	0.572	0.614	0.629
		None	0.150	0.619	0.695	0.573	0.613	0.628
	ESMc	NCR	0.148	0.618	0.693	0.572	0.597	0.596
		None	0.151	0.612	0.671	0.574	0.600	0.597
cVAEResDNN	ESM2	NCRENN	0.228	0.625	0.643	0.614	0.646	0.626
		NCR	0.217	0.658	0.763	0.603	0.631	0.596
		None	0.202	0.608	0.617	0.601	0.636	0.608
	ESMc	NCRENN	0.168	0.604	0.636	0.584	0.622	0.599
		NCR	0.200	0.627	0.674	0.599	0.608	0.568
		None	0.162	0.572	0.559	0.581	0.610	0.563
cVAEDNNLiner	ESM2	NCRENN	0.205	0.623	0.656	0.602	0.622	0.591
		NCR	0.231	0.660	0.757	0.610	0.621	0.578
		None	0.231	0.616	0.616	0.615	0.649	0.621
	ESMc	NCRENN	0.185	0.618	0.659	0.592	0.618	0.578
		NCR	0.179	0.614	0.653	0.589	0.615	0.574
		None	0.203	0.614	0.634	0.601	0.613	0.560
