# Supplementary material for: EUP: Enhanced cross-species prediction of ubiquitination sites via a conditional variational autoencoder network based on ESM2
Source: PLoS Comput Biol. 2025 Jul 16;21(7):e1013268. doi: 10.1371/journal.pcbi.1013268 (PMC12266453; doi:10.1371/journal.pcbi.1013268)
Supplement: S6 Table — (PDF) [file pcbi.1013268.s013.pdf]

S6 Table. Performance of Optimal Models for Single Species Data

specie	model_name	MCC	F1	Recall	Accuracy	AUC	PR
Arabidopsis	10species[ResDNNModel_10species]	0.094	0.153	0.299	0.823	0.644	0.096
Arabidopsis	10species[DNNLinerModel_10species]	0.084	0.144	0.333	0.789	0.634	0.091
Arabidopsis	10species[CVAE_ResDNNModel_10species]	0.098	0.155	0.211	0.877	0.651	0.099
Arabidopsis	10species[CVAE_DNNLinerModel_10species]	0.094	0.147	0.163	0.899	0.630	0.100
Arabidopsis	10species[CVAE_ResDNNModel_10species_NCR]	0.099	0.157	0.236	0.865	0.656	0.098
Arabidopsis	10species[CVAE_ResDNNModel_10species_NCREN]	0.090	0.148	0.364	0.776	0.643	0.089
Arabidopsis	10species[CVAE_DNNLinerModel_10species_NCR]	0.113	0.169	0.274	0.856	0.607	0.089
Arabidopsis	10species[CVAE_DNNLinerModel_10species_NCREN]	0.100	0.154	0.388	0.772	0.634	0.089
Arabidopsis	Arabidopsis[ResDNNModel_Arabidopsis]	0.204	0.251	0.331	0.894	0.749	0.182
Arabidopsis	Arabidopsis[DNNLinerModel_Arabidopsis]	0.208	0.232	0.548	0.807	0.762	0.177
Arabidopsis	Arabidopsis[CVAE_ResDNNModel_Arabidopsis]	0.132	0.121	0.075	0.942	0.684	0.138
Arabidopsis	Arabidopsis[CVAE_DNNLinerModel_Arabidopsis]	0.156	0.173	0.125	0.936	0.651	0.136
Arabidopsis	Arabidopsis[CVAE_ResDNNModel_Arabidopsis_NCR]	0.163	0.172	0.121	0.938	0.675	0.147
Arabidopsis	Arabidopsis[CVAE_ResDNNModel_Arabidopsis_NCREN]	0.159	0.184	0.144	0.932	0.708	0.151
Arabidopsis	Arabidopsis[CVAE_DNNLinerModel_Arabidopsis_NCR]	0.153	0.177	0.135	0.933	0.659	0.140
Arabidopsis	Arabidopsis[CVAE_DNNLinerModel_Arabidopsis_NCREN]	0.166	0.196	0.160	0.930	0.665	0.145
Candida	10species[ResDNNModel_10species]	0.128	0.165	0.588	0.672	0.701	0.158
Candida	10species[DNNLinerModel_10species]	0.078	0.134	0.588	0.581	0.625	0.187
Candida	10species[CVAE_ResDNNModel_10species]	0.030	0.110	0.294	0.737	0.554	0.140
Candida	10species[CVAE_DNNLinerModel_10species]	0.110	0.167	0.353	0.805	0.629	0.110
Candida	10species[CVAE_ResDNNModel_10species_NCR]	0.045	0.120	0.353	0.714	0.601	0.132
Candida	10species[CVAE_ResDNNModel_10species_NCREN]	0.112	0.151	0.647	0.597	0.699	0.110
Candida	10species[CVAE_DNNLinerModel_10species_NCR]	0.105	0.157	0.471	0.721	0.629	0.117
Candida	10species[CVAE_DNNLinerModel_10species_NCREN]	0.124	0.158	0.647	0.620	0.604	0.078
Candida	Candida[ResDNNModel_Candida]	0.296	0.308	0.235	0.942	0.598	0.240
Candida	Candida[DNNLinerModel_Candida]	0.078	0.143	0.294	0.805	0.652	0.104
Candida	Candida[CVAE_ResDNNModel_Candida]	0.194	0.182	0.118	0.942	0.657	0.231
Candida	Candida[CVAE_DNNLinerModel_Candida]	0.052	0.108	0.118	0.893	0.687	0.130
Candida	Candida[CVAE_ResDNNModel_Candida_NCR]	-0.020	0.000	0.000	0.938	0.469	0.055
Candida	Candida[CVAE_ResDNNModel_Candida_NCREN]	-0.028	0.000	0.000	0.932	0.621	0.111
Candida	Candida[CVAE_DNNLinerModel_Candida_NCR]	0.092	0.154	0.294	0.821	0.657	0.123
Candida	Candida[CVAE_DNNLinerModel_Candida_NCREN]	0.008	0.080	0.118	0.851	0.684	0.120
Drosophila	10species[ResDNNModel_10species]	0.211	0.264	0.453	0.833	0.749	0.206
Drosophila	10species[DNNLinerModel_10species]	0.152	0.209	0.480	0.760	0.695	0.142
Drosophila	10species[CVAE_ResDNNModel_10species]	0.291	0.340	0.442	0.887	0.757	0.364
Drosophila	10species[CVAE_DNNLinerModel_10species]	0.311	0.360	0.409	0.904	0.742	0.344
Drosophila	10species[CVAE_ResDNNModel_10species_NCR]	0.267	0.313	0.493	0.857	0.764	0.244
Drosophila	10species[CVAE_ResDNNModel_10species_NCREN]	0.232	0.259	0.650	0.755	0.772	0.219
Drosophila	10species[CVAE_DNNLinerModel_10species_NCR]	0.262	0.307	0.496	0.852	0.741	0.243

Drosophila	10species[CVAE_DNNLinerModel_10species_NCREN]	0.227	0.257	0.636	0.757	0.762	0.203
Drosophila	Drosophila[ResDNNModel_Drosophila]	0.180	0.234	0.469	0.797	0.722	0.189
Drosophila	Drosophila[DNNLinerModel_Drosophila]	0.193	0.240	0.521	0.782	0.736	0.191
Drosophila	Drosophila[CVAE_ResDNNModel_Drosophila]	0.143	0.126	0.076	0.930	0.656	0.162
Drosophila	Drosophila[CVAE_DNNLinerModel_Drosophila]	0.125	0.148	0.105	0.920	0.628	0.141
Drosophila	Drosophila[CVAE_ResDNNModel_Drosophila_NCR]	0.171	0.188	0.134	0.924	0.679	0.180
Drosophila	Drosophila[CVAE_ResDNNModel_Drosophila_NCREN]	0.161	0.194	0.152	0.917	0.680	0.174
Drosophila	Drosophila[CVAE_DNNLinerModel_Drosophila_NCR]	0.145	0.168	0.121	0.921	0.627	0.153
Drosophila	Drosophila[CVAE_DNNLinerModel_Drosophila_NCREN]	0.151	0.204	0.196	0.899	0.654	0.166
Emericella	10species[ResDNNModel_10species]	0.219	0.290	0.404	0.841	0.742	0.227
Emericella	10species[DNNLinerModel_10species]	0.214	0.283	0.461	0.812	0.746	0.222
Emericella	10species[CVAE_ResDNNModel_10species]	0.171	0.245	0.285	0.859	0.691	0.197
Emericella	10species[CVAE_DNNLinerModel_10species]	0.183	0.250	0.255	0.877	0.673	0.189
Emericella	10species[CVAE_ResDNNModel_10species_NCR]	0.188	0.263	0.335	0.849	0.700	0.188
Emericella	10species[CVAE_ResDNNModel_10species_NCREN]	0.193	0.259	0.540	0.751	0.724	0.190
Emericella	10species[CVAE_DNNLinerModel_10species_NCR]	0.178	0.255	0.349	0.836	0.663	0.165
Emericella	10species[CVAE_DNNLinerModel_10species_NCREN]	0.198	0.263	0.538	0.758	0.706	0.177
Emericella	Emericella[ResDNNModel_Emericella]	0.230	0.300	0.379	0.858	0.741	0.253
Emericella	Emericella[DNNLinerModel_Emericella]	0.228	0.284	0.583	0.764	0.757	0.247
Emericella	Emericella[CVAE_ResDNNModel_Emericella]	0.159	0.173	0.117	0.910	0.704	0.205
Emericella	Emericella[CVAE_DNNLinerModel_Emericella]	0.175	0.214	0.166	0.902	0.659	0.195
Emericella	Emericella[CVAE_ResDNNModel_Emericella_NCR]	0.203	0.252	0.214	0.898	0.688	0.226
Emericella	Emericella[CVAE_ResDNNModel_Emericella_NCREN]	0.193	0.253	0.238	0.887	0.687	0.215
Emericella	Emericella[CVAE_DNNLinerModel_Emericella_NCR]	0.194	0.249	0.222	0.892	0.657	0.211
Emericella	Emericella[CVAE_DNNLinerModel_Emericella_NCREN]	0.185	0.251	0.253	0.879	0.673	0.203
Homo	10species[ResDNNModel_10species]	0.259	0.552	0.600	0.642	0.687	0.542
Homo	10species[DNNLinerModel_10species]	0.267	0.567	0.651	0.635	0.692	0.542
Homo	10species[CVAE_ResDNNModel_10species]	0.270	0.466	0.379	0.681	0.687	0.566
Homo	10species[CVAE_DNNLinerModel_10species]	0.239	0.426	0.333	0.671	0.669	0.548
Homo	10species[CVAE_ResDNNModel_10species_NCR]	0.282	0.505	0.442	0.681	0.689	0.576
Homo	10species[CVAE_ResDNNModel_10species_NCREN]	0.306	0.583	0.645	0.661	0.710	0.580
Homo	10species[CVAE_DNNLinerModel_10species_NCR]	0.288	0.512	0.454	0.683	0.673	0.566
Homo	10species[CVAE_DNNLinerModel_10species_NCREN]	0.302	0.578	0.632	0.662	0.697	0.562
Homo	Homo[ResDNNModel_Homo]	0.371	0.622	0.692	0.691	0.757	0.619
Homo	Homo[DNNLinerModel_Homo]	0.368	0.627	0.735	0.679	0.754	0.614
Homo	Homo[CVAE_ResDNNModel_Homo]	0.389	0.621	0.645	0.711	0.764	0.632
Homo	Homo[CVAE_DNNLinerModel_Homo]	0.385	0.617	0.635	0.710	0.753	0.621
Homo	Homo[CVAE_ResDNNModel_Homo_NCR]	0.393	0.644	0.783	0.682	0.767	0.623
Homo	Homo[CVAE_ResDNNModel_Homo_NCREN]	0.370	0.636	0.842	0.647	0.752	0.596
Homo	Homo[CVAE_DNNLinerModel_Homo_NCR]	0.388	0.638	0.746	0.690	0.751	0.596
Homo	Homo[CVAE_DNNLinerModel_Homo_NCREN]	0.363	0.633	0.830	0.646	0.741	0.571
Mus	10species[ResDNNModel_10species]	0.174	0.247	0.664	0.636	0.705	0.193

Mus	10species[DNNLinerModel_10species]	0.156	0.233	0.682	0.596	0.684	0.177
Mus	10species[CVAE_ResDNNModel_10species]	0.202	0.278	0.548	0.745	0.700	0.209
Mus	10species[CVAE_DNNLinerModel_10species]	0.206	0.283	0.514	0.767	0.697	0.208
Mus	10species[CVAE_ResDNNModel_10species_NCR]	0.203	0.273	0.612	0.708	0.710	0.205
Mus	10species[CVAE_ResDNNModel_10species_NCREN]	0.187	0.248	0.749	0.593	0.718	0.198
Mus	10species[CVAE_DNNLinerModel_10species_NCR]	0.195	0.267	0.606	0.702	0.693	0.188
Mus	10species[CVAE_DNNLinerModel_10species_NCREN]	0.185	0.247	0.739	0.596	0.703	0.177
Mus	Mus[ResDNNModel_Mus]	0.215	0.294	0.355	0.847	0.730	0.235
Mus	Mus[DNNLinerModel_Mus]	0.241	0.308	0.575	0.768	0.755	0.245
Mus	Mus[CVAE_ResDNNModel_Mus]	0.185	0.213	0.153	0.899	0.706	0.232
Mus	Mus[CVAE_DNNLinerModel_Mus]	0.187	0.254	0.239	0.874	0.676	0.207
Mus	Mus[CVAE_ResDNNModel_Mus_NCR]	0.211	0.277	0.263	0.877	0.717	0.239
Mus	Mus[CVAE_ResDNNModel_Mus_NCREN]	0.219	0.289	0.288	0.873	0.724	0.235
Mus	Mus[CVAE_DNNLinerModel_Mus_NCR]	0.198	0.246	0.198	0.891	0.681	0.228
Mus	Mus[CVAE_DNNLinerModel_Mus_NCREN]	0.204	0.276	0.280	0.868	0.685	0.221
Oryza	10species[ResDNNModel_10species]	0.320	0.364	0.595	0.840	0.812	0.334
Oryza	10species[DNNLinerModel_10species]	0.156	0.230	0.437	0.775	0.685	0.157
Oryza	10species[CVAE_ResDNNModel_10species]	0.597	0.625	0.741	0.932	0.900	0.725
Oryza	10species[CVAE_DNNLinerModel_10species]	0.639	0.667	0.715	0.945	0.895	0.720
Oryza	10species[CVAE_ResDNNModel_10species_NCR]	0.503	0.525	0.759	0.895	0.890	0.498
Oryza	10species[CVAE_ResDNNModel_10species_NCREN]	0.394	0.399	0.830	0.807	0.884	0.417
Oryza	10species[CVAE_DNNLinerModel_10species_NCR]	0.516	0.536	0.784	0.896	0.883	0.493
Oryza	10species[CVAE_DNNLinerModel_10species_NCREN]	0.389	0.394	0.826	0.804	0.877	0.413
Oryza	Oryza[ResDNNModel_Oryza]	0.225	0.232	0.159	0.919	0.720	0.239
Oryza	Oryza[DNNLinerModel_Oryza]	0.211	0.270	0.536	0.777	0.739	0.216
Oryza	Oryza[CVAE_ResDNNModel_Oryza]	0.177	0.187	0.126	0.916	0.649	0.209
Oryza	Oryza[CVAE_DNNLinerModel_Oryza]	0.179	0.207	0.153	0.910	0.648	0.206
Oryza	Oryza[CVAE_ResDNNModel_Oryza_NCR]	0.196	0.224	0.166	0.911	0.686	0.221
Oryza	Oryza[CVAE_ResDNNModel_Oryza_NCREN]	0.185	0.229	0.187	0.903	0.682	0.207
Oryza	Oryza[CVAE_DNNLinerModel_Oryza_NCR]	0.198	0.240	0.196	0.905	0.661	0.218
Oryza	Oryza[CVAE_DNNLinerModel_Oryza_NCREN]	0.191	0.250	0.237	0.890	0.668	0.213
Rattus	10species[ResDNNModel_10species]	0.146	0.181	0.638	0.657	0.692	0.135
Rattus	10species[DNNLinerModel_10species]	0.102	0.154	0.616	0.599	0.655	0.103
Rattus	10species[CVAE_ResDNNModel_10species]	0.203	0.230	0.597	0.764	0.731	0.183
Rattus	10species[CVAE_DNNLinerModel_10species]	0.201	0.234	0.553	0.786	0.725	0.181
Rattus	10species[CVAE_ResDNNModel_10species_NCR]	0.190	0.214	0.644	0.720	0.735	0.141
Rattus	10species[CVAE_ResDNNModel_10species_NCREN]	0.157	0.179	0.734	0.603	0.718	0.133
Rattus	10species[CVAE_DNNLinerModel_10species_NCR]	0.184	0.210	0.634	0.717	0.706	0.127
Rattus	10species[CVAE_DNNLinerModel_10species_NCREN]	0.167	0.185	0.747	0.611	0.722	0.127
Rattus	Rattus[ResDNNModel_Rattus]	0.202	0.213	0.152	0.933	0.703	0.202
Rattus	Rattus[DNNLinerModel_Rattus]	0.194	0.230	0.537	0.787	0.737	0.191
Rattus	Rattus[CVAE_ResDNNModel_Rattus]	0.185	0.162	0.100	0.939	0.661	0.175

Rattus	Rattus[CVAE_DNNLinerModel_Rattus]	0.171	0.171	0.113	0.935	0.611	0.166
Rattus	Rattus[CVAE_ResDNNModel_Rattus_NCR]	0.197	0.197	0.134	0.936	0.659	0.174
Rattus	Rattus[CVAE_ResDNNModel_Rattus_NCREN]	0.187	0.201	0.145	0.932	0.673	0.186
Rattus	Rattus[CVAE_DNNLinerModel_Rattus_NCR]	0.209	0.218	0.155	0.934	0.639	0.186
Rattus	Rattus[CVAE_DNNLinerModel_Rattus_NCREN]	0.183	0.213	0.170	0.926	0.645	0.174
Saccharomy	10species[ResDNNModel_10species]	0.220	0.336	0.371	0.798	0.702	0.305
Saccharomy	10species[DNNLinerModel_10species]	0.198	0.321	0.366	0.786	0.692	0.279
Saccharomy	10species[CVAE_ResDNNModel_10species]	0.236	0.320	0.274	0.840	0.690	0.314
Saccharomy	10species[CVAE_DNNLinerModel_10species]	0.221	0.283	0.217	0.849	0.659	0.297
Saccharomy	10species[CVAE_ResDNNModel_10species_NCR]	0.248	0.334	0.292	0.840	0.705	0.323
Saccharomy	10species[CVAE_ResDNNModel_10species_NCREN]	0.243	0.365	0.532	0.745	0.721	0.322
Saccharomy	10species[CVAE_DNNLinerModel_10species_NCR]	0.234	0.341	0.348	0.815	0.669	0.300
Saccharomy	10species[CVAE_DNNLinerModel_10species_NCREN]	0.245	0.366	0.496	0.763	0.697	0.296
Saccharomy	Saccharomyces[ResDNNModel_Saccharomyces]	0.309	0.386	0.335	0.853	0.756	0.395
Saccharomy	Saccharomyces[DNNLinerModel_Saccharomyces]	0.291	0.400	0.603	0.751	0.762	0.377
Saccharomy	Saccharomyces[CVAE_ResDNNModel_Saccharomyces]	0.301	0.345	0.257	0.865	0.741	0.389
Saccharomy	Saccharomyces[CVAE_DNNLinerModel_Saccharomyces]	0.284	0.358	0.299	0.852	0.711	0.370
Saccharomy	Saccharomyces[CVAE_ResDNNModel_Saccharomyces_N	0.313	0.393	0.348	0.852	0.744	0.389
Saccharomy	Saccharomyces[CVAE_ResDNNModel_Saccharomyces_N	0.303	0.411	0.515	0.797	0.753	0.369
Saccharomy	Saccharomyces[CVAE_DNNLinerModel_Saccharomyces_	0.310	0.402	0.393	0.839	0.724	0.378
Saccharomy	Saccharomyces[CVAE_DNNLinerModel_Saccharomyces_	0.282	0.395	0.508	0.785	0.738	0.358
Toxoplasma	10species[ResDNNModel_10species]	0.146	0.204	0.347	0.837	0.720	0.144
Toxoplasma	10species[DNNLinerModel_10species]	0.124	0.182	0.421	0.773	0.697	0.123
Toxoplasma	10species[CVAE_ResDNNModel_10species]	0.162	0.220	0.314	0.866	0.695	0.165
Toxoplasma	10species[CVAE_DNNLinerModel_10species]	0.143	0.199	0.225	0.892	0.642	0.134
Toxoplasma	10species[CVAE_ResDNNModel_10species_NCR]	0.130	0.191	0.310	0.842	0.701	0.124
Toxoplasma	10species[CVAE_ResDNNModel_10species_NCREN]	0.162	0.207	0.491	0.775	0.714	0.129
Toxoplasma	10species[CVAE_DNNLinerModel_10species_NCR]	0.148	0.205	0.354	0.836	0.676	0.127
Toxoplasma	10species[CVAE_DNNLinerModel_10species_NCREN]	0.158	0.201	0.535	0.744	0.699	0.119
Toxoplasma	Toxoplasma[ResDNNModel_Toxoplasma]	0.161	0.216	0.251	0.891	0.711	0.164
Toxoplasma	Toxoplasma[DNNLinerModel_Toxoplasma]	0.193	0.232	0.524	0.791	0.757	0.172
Toxoplasma	Toxoplasma[CVAE_ResDNNModel_Toxoplasma]	0.130	0.169	0.140	0.917	0.698	0.154
Toxoplasma	Toxoplasma[CVAE_DNNLinerModel_Toxoplasma]	0.150	0.166	0.118	0.929	0.654	0.144
Toxoplasma	Toxoplasma[CVAE_ResDNNModel_Toxoplasma_NCR]	0.147	0.194	0.181	0.910	0.679	0.150
Toxoplasma	Toxoplasma[CVAE_ResDNNModel_Toxoplasma_NCREN]	0.104	0.131	0.096	0.924	0.703	0.151
Toxoplasma	Toxoplasma[CVAE_DNNLinerModel_Toxoplasma_NCR]	0.124	0.165	0.140	0.915	0.669	0.139
Toxoplasma	Toxoplasma[CVAE_DNNLinerModel_Toxoplasma_NCREN	0.127	0.174	0.159	0.909	0.653	0.148
